# Supplementary material for: Macroscopic homochiral helicoids self-assembled via screw dislocations
Source: Nat Commun. 2024 Jul 24;15:6233. doi: 10.1038/s41467-024-50631-3 (PMC11266591; doi:10.1038/s41467-024-50631-3)
Supplement: Supplementary file 1 — Supplementary Information [file 41467_2024_50631_MOESM1_ESM.pdf]

Supplementary Information

Macroscopic Homochiral Helicoids Self-Assembled via Screw Dislocations

Shengfu Wu[1,2], Xin Song[1], Cong Du[1] & Minghua Liu[1,2]*

[1]Beijing National Laboratory of Molecular Sciences (BNLMS) and CAS Key Laboratory of Colloid, Interface and Thermodynamics, Institute of Chemistry, Chinese Academy of Sciences; North First Street 2, Zhongguancun, Beijing, 100190, China.

[2]University of Chinese Academy of Sciences; No.19(A) Yuquan Road, Beijing, 100049, China.

*Corresponding to: liumh@iccas.ac.cn

Table of Content

1. Supplementary synthetic procedures 3

2. Supplementary diagram of co-assembly 4

3. Supplementary photographs 5

4. Supplementary SEM, AFM and TEM images 6

5. Supplementary single crystal data 19

6. Supplementary density functional theory calculations 27

7. Supplementary spectra 28

8. Supplementary ^{1}H-NMR spectra of helicoids 33

9. Supplementary XRD patterns 35

10. Supplementary NMR and MS spectra 40

11. Supplementary references 43

1. Supplementary synthetic procedures

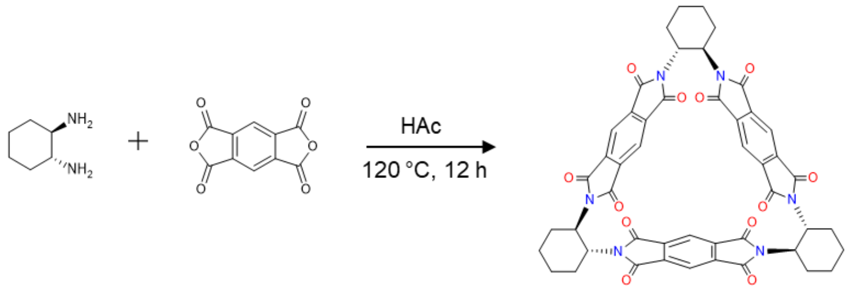

(1*R*,2*R*)-1,2-Diaminocyclohexane (*RRRRRR*)-PMDI-Δ (*R*-Δ)

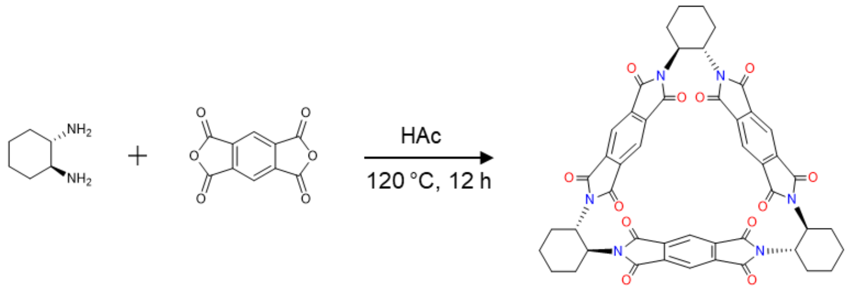

(1*S*,2*S*)-1,2-Diaminocyclohexane (*SSSSSS*)-PMDI-Δ (*S*-Δ)

Supplementary Figure 1. Synthesis routes to *R*-PMDI-Δ and *S*-PMDI-Δ.

R-PMDI-Δ and *S*-PMDI-Δ were synthesized following the supplementary reference 1, and the post-treatment methods of the reactions were slightly different.

Compound *R*-PMDI-Δ: Pyromellitic dianhydride (PMDA, 5.0 g, 22.9 mmol) was added to a 500 mL round-bottomed flask containing 225 mL acetic acid (HAc). The mixture was stirred for about 15 min at room temperature, (*RR*)-1,2-cyclohexanediamine (2.61 g, 22.9 mmol) was added in four portions within the next 1 hour. Then the reaction mixture was refluxed at 120°C for 12 h. Subsequently, the reaction mixture was cooled and concentrated in vacuo at 60°C to remove HAc. Next, 300 mL DCM was added and the mixture was stirred at room temperature for 2 hours. Then, the mixture was filtered and the filtrate was collected. The filtrate was concentrated to approximately 20 mL. Subsequently, 20 mL ethyl acetate (EtOAc) was dropped into the concentrated filtrate and a white solid precipitated. The precipitate was collected by filtration, and the residual solvent in the solid was removed by vacuum drying. Finally, 2.0 g of white solids were obtained, yielding 30%.

HR-ESI-MS: calc: $C_{48}H_{36}N_6O_{12}$ = 888.24, found: 911.227810 $[M + Na]^+$.

^{1}H NMR (400 MHz, $CDCl_3$) δ 8.03 (s, 6H), 5.19-5.17 (m, 6H), 2.10 (m, 12H), 1.90 (m, 6H), 1.57

(m, 6H). ^{13}C NMR (100 MHz, $CDCl_3$) δ 165.6, 164.5, 136.5, 136.2, 118.9, 51.2, 30.9, 25.0.

Compound *S*-PMDI-Δ: The synthetic procedures of *S*-PMDI-Δ enantiomer are the same with that of *R*-PMDI-Δ.

HR-ESI-MS: calc: $C_{48}H_{36}N_6O_{12}$ = 888.24, found: 911.228282 $[M + Na]^+$.

^1H NMR (400 MHz, $CDCl_3$) δ 8.03 (s, 6H), 5.19 – 5.16 (m, 6H), 2.10 (m, 12H), 1.93-1.90 (m, 6H), 1.60-1.57 (m, 6H). ^{13}C NMR (100 MHz, $CDCl_3$) δ 165.6, 164.9, 136.4, 136.2, 118.9, 51.2, 30.9, 25.0.

2. Supplementary diagram of co-assembly

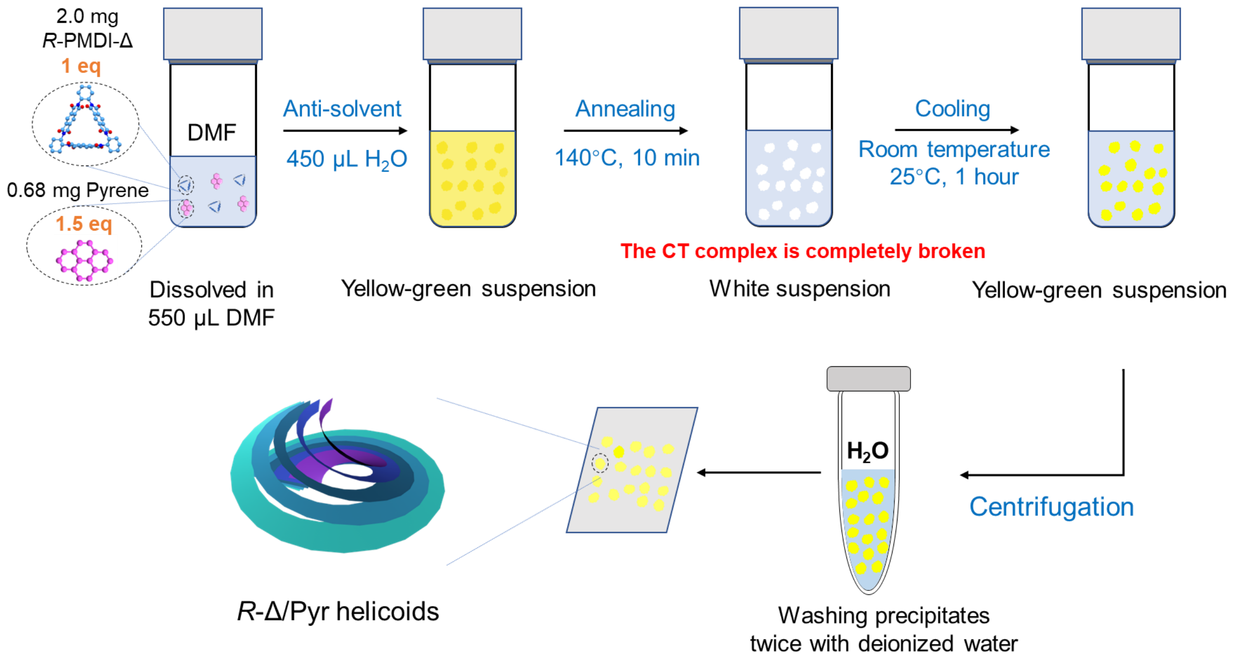

Supplementary Figure 2. Diagram of co-assembly protocol of *R*-PMDI-Δ/Pyr helicoids in DMF/H_2O mixed solvent.

3. Supplementary photographs

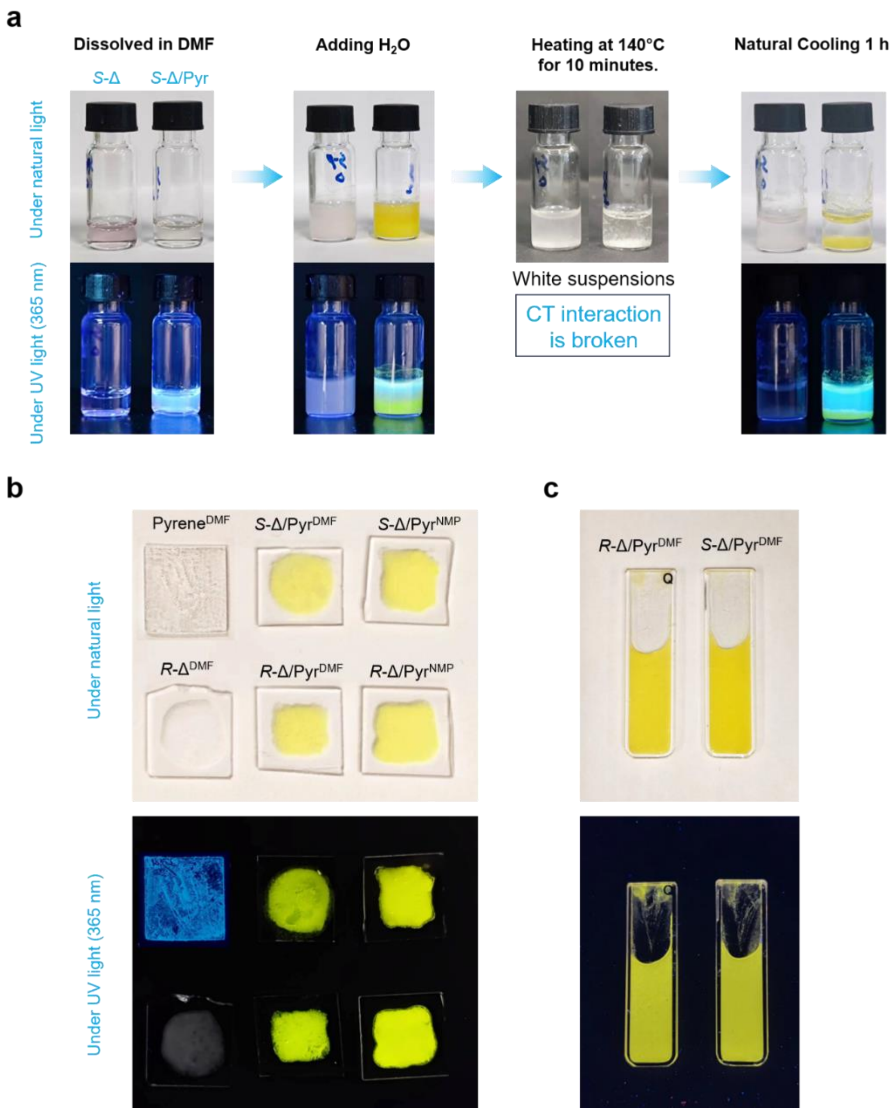

Supplementary Figure 3. (**a**) Photographs of sample vials at different assembly stages under natural light and UV light. (**b**) Photographs of individual PMDI-Δ assemblies^{DMF}, pyrene powder and PMDI-Δ/Pyr co-assemblies under natural and 365 nm UV light after annealing. The aqueous solution of the samples was dispersed on the quartz wafer with the size about 1 cm × 1 cm and naturally dried at room temperature. (**c**) Photographs of PMDI-Δ/Pyr co-assemblies^{DMF} under natural light and UV light after annealing dispersed in deionized water in a 1 mm quartz cuvette.

4. Supplementary SEM, AFM and TEM images

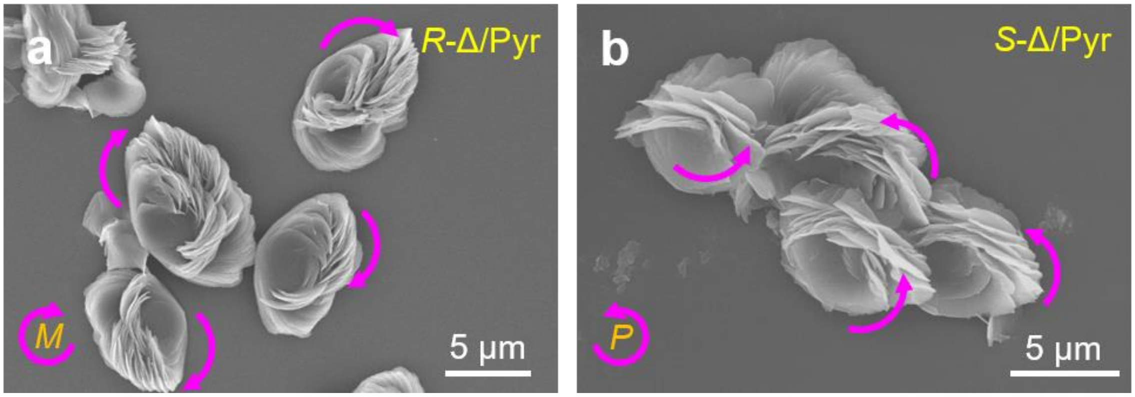

Supplementary Figure 4. SEM images of *R*-PMDI-Δ/Pyr helicoids^{DMF} (**a**) and *S*-PMDI-Δ/Pyr helicoids^{DMF} (**b**) obtained after annealing according to Supplementary Figure 1.

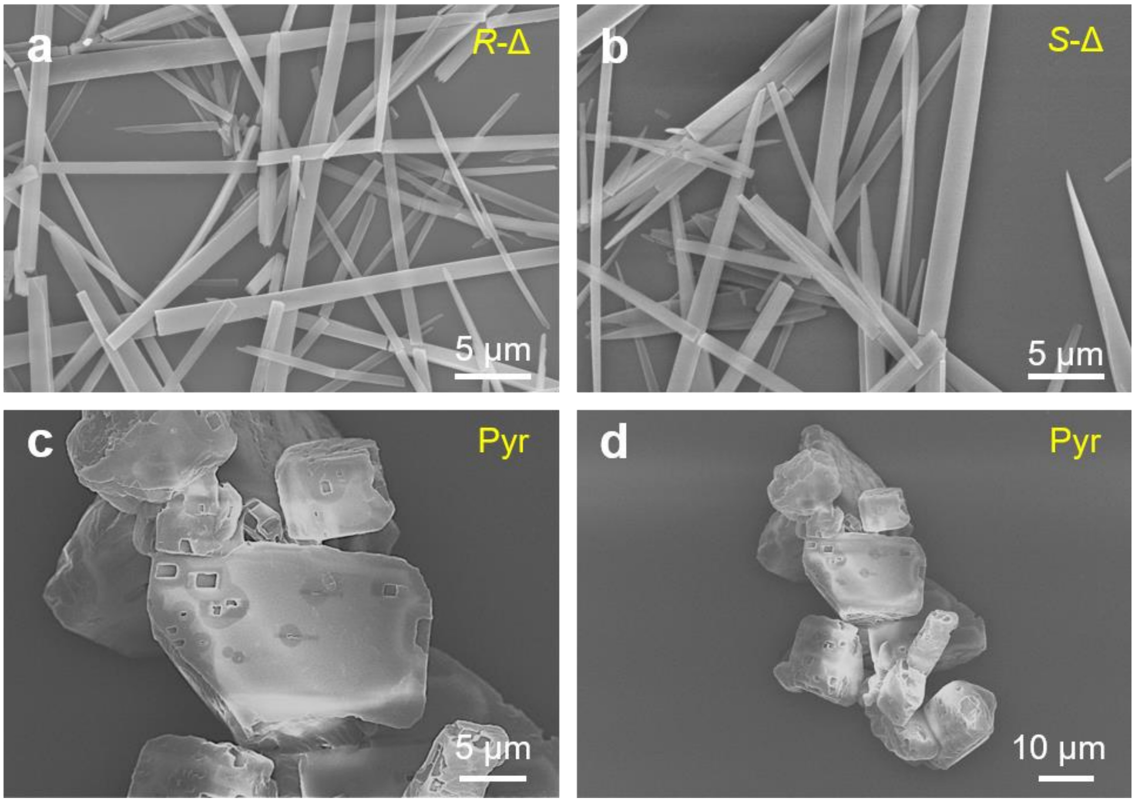

Supplementary Figure 5. SEM images of individual *R*-PMDI-Δ assemblies^{DMF} (**a**), *S*-PMDI-Δ assemblies^{DMF} (**b**) and pyrene powder^{DMF} (**c** and **d**) obtained after annealing. (Preparation method of pyrene powder^{DMF}: Pyrene 1.0 mg, DMF/H_2O *v*/*v* =200 μL/200 μL).

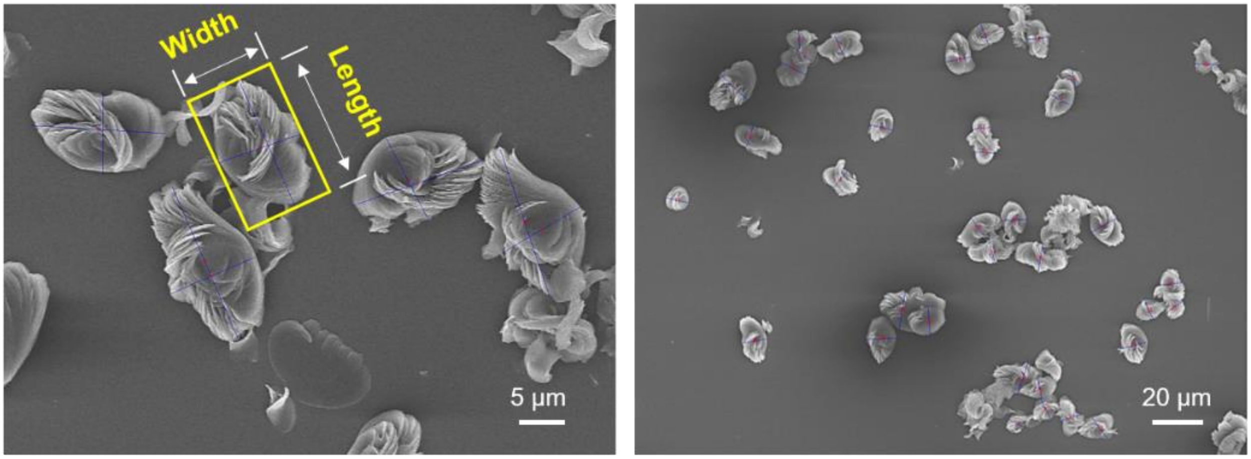

Supplementary Figure 6. Statistical analysis of the length and width of *R*-PMDI-Δ/Pyr helicoids^{DMF}. The length and width (white lines) are measured with a Nano measurer software.

Supplementary Table 1.

The bottom left table and the bottom right table summarize the distributions of the length and width of helicoids in Supplementary Figure 6 respectively.

Length (µm)

Length (µm)	Counts	Percentage
8-10	2	4.9%
10-12	3	7.3%
12-14	6	14.6%
14-16	12	29.3%
16-18	7	17.1%
18-20	7	17.1%
20-22	2	4.9%
22-24	1	2.4%
24-26	1	2.4%

Width (µm)

Width (µm)	Counts	Percentage
6-8	5	12.2%
8-10	23	56.1%
10-12	9	22.0%
12-14	2	4.9%
14-16	2	4.9%

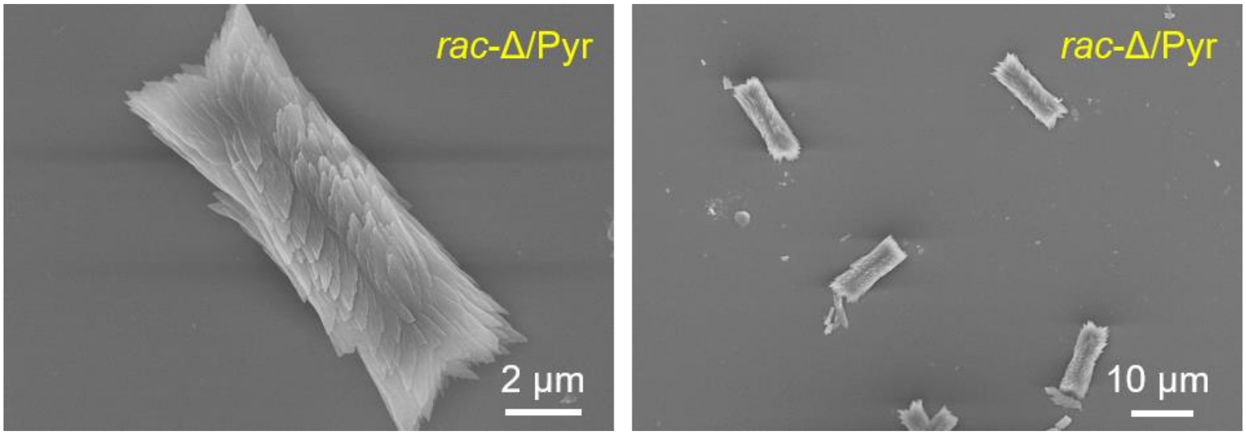

Supplementary Figure 7. SEM image of *rac*-PMDI-Δ/Pyr co-assemblies^{DMF} obtained after annealing.

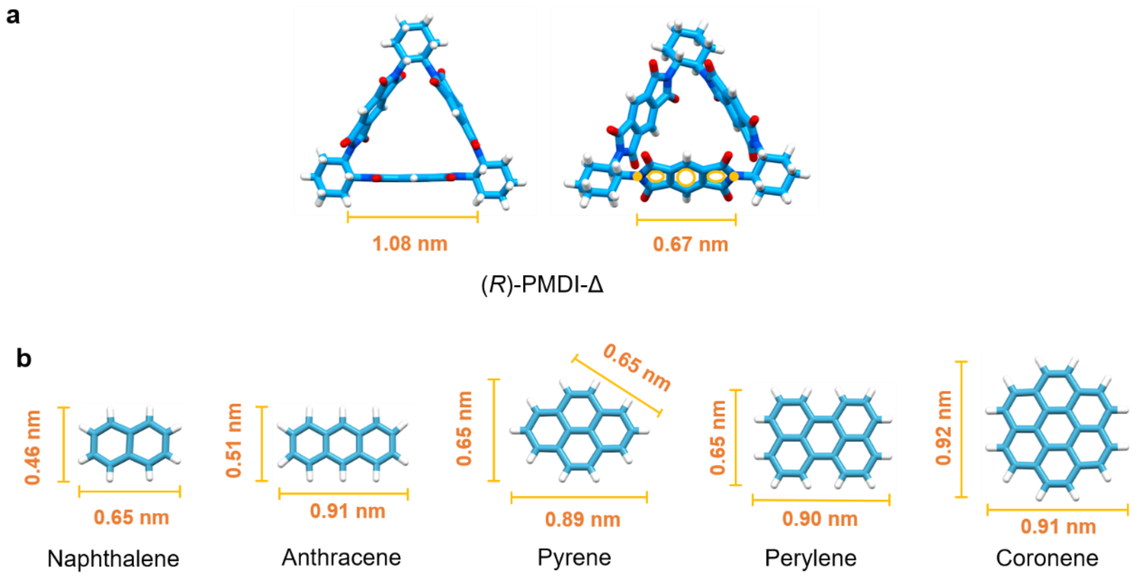

Supplementary Figure 8. (**a**) The distance between H atoms on the cyclohexyl group of the *R*-PMDI-Δ molecule and the length of the π-conjugated surface on one side of the *R*-PMDI-Δ molecule. (**b**) The size of several common electron-rich polycyclic aromatic hydrocarbon molecules. The data was taken from CCDC numbers 1216808 (naphthalene), 1103062 (anthracene), 118728 (pyrene), 1231184 (perylene), and 1129883 (coronene).

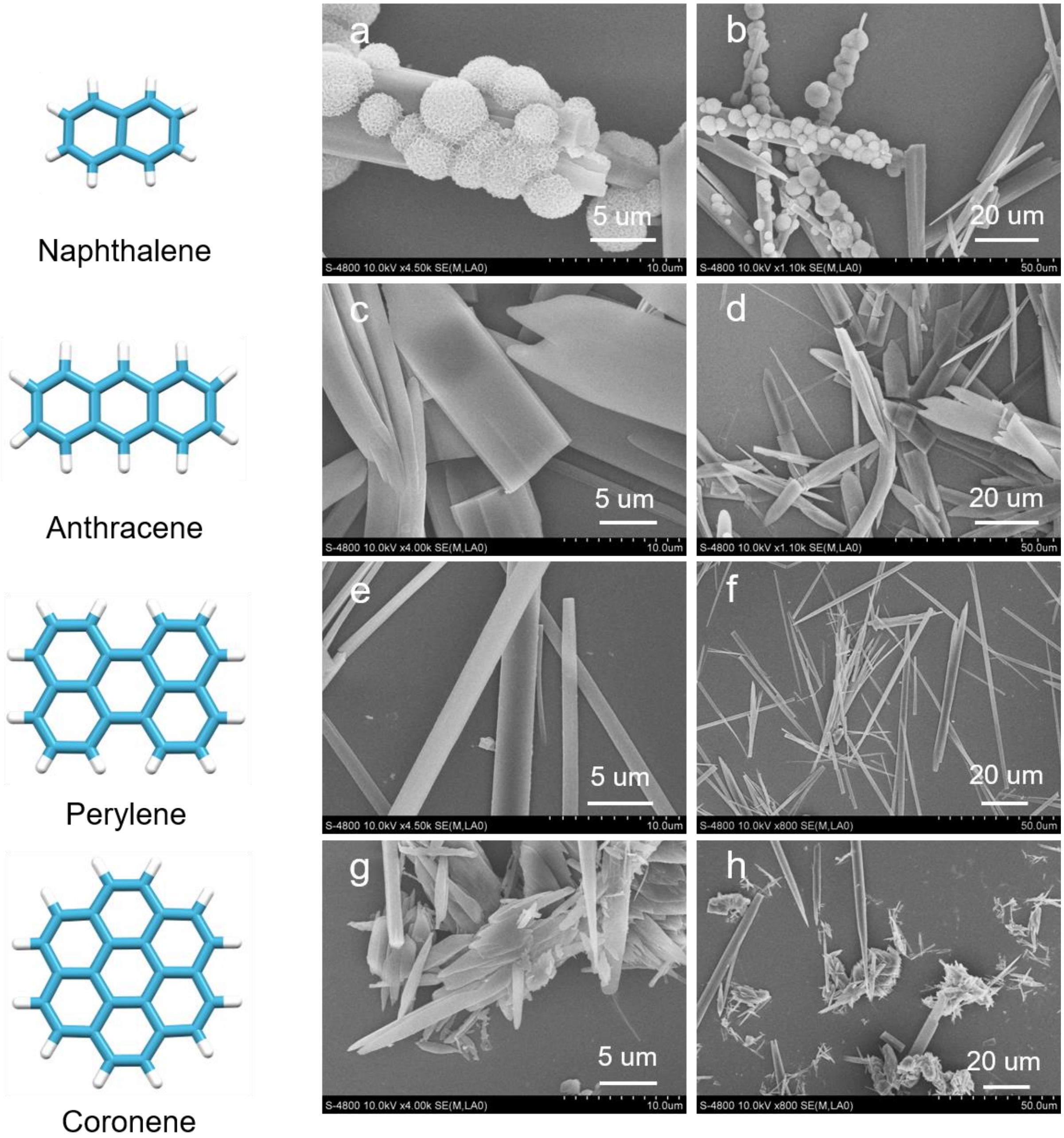

Supplementary Figure 9. SEM images of different combination co-assemblies obtained in DMF/H_2O under the same co-assembly protocol as in Supplementary Fig. 2, except that the two molar equivalent electron donors relative to *R*-PMDI-Δ were added. (**a** and **b**) *R*-PMDI-Δ/Naphthalene. (**c** and **d**) *R*-PMDI-Δ/Anthracene. (**e** and **f)** *R*-PMDI-Δ/Perylene. (**g** and **h**) *R*-PMDI-Δ/Coronene. The experimental results showed that none of these electron donors with different sizes can form homochiral helicoids with *R*-PMDI-Δ, indicating a special size matching between PMDI-Δ and pyrene.

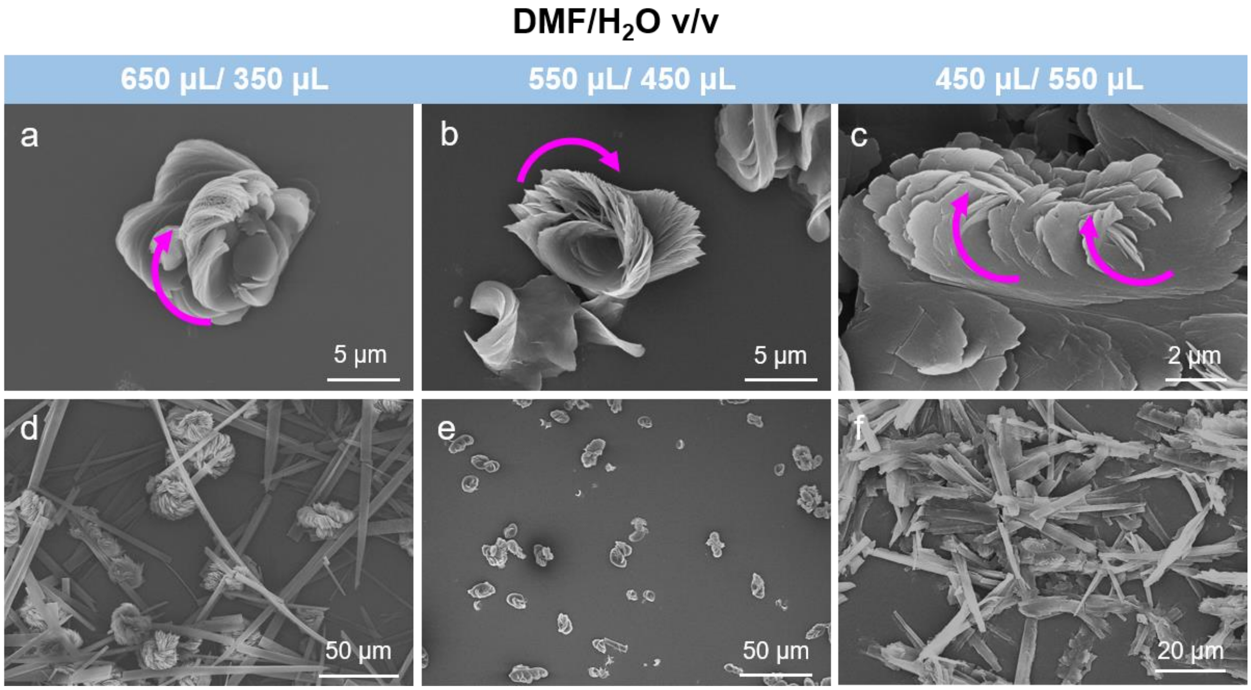

Supplementary Figure 10. SEM image of *R*-Δ/Pyr co-assemblies in different volume ratios of DMF to H_2O. DMF/H_2O *v*/*v* = 650 µL/350 µL (**a** and **d**), 550 µL/450 µL (**b** and **e**), 450 µL/550 µL (**c** and **f**). The macroscopic morphology of the co-assemblies showed sensitivity to solvent volume ratio. Both the high DMF/H_2O solvent ratio (65%/35%) and the low DMF/H_2O solvent ratio (45%/55%) are not in favor of the formation of uniform helicoids.

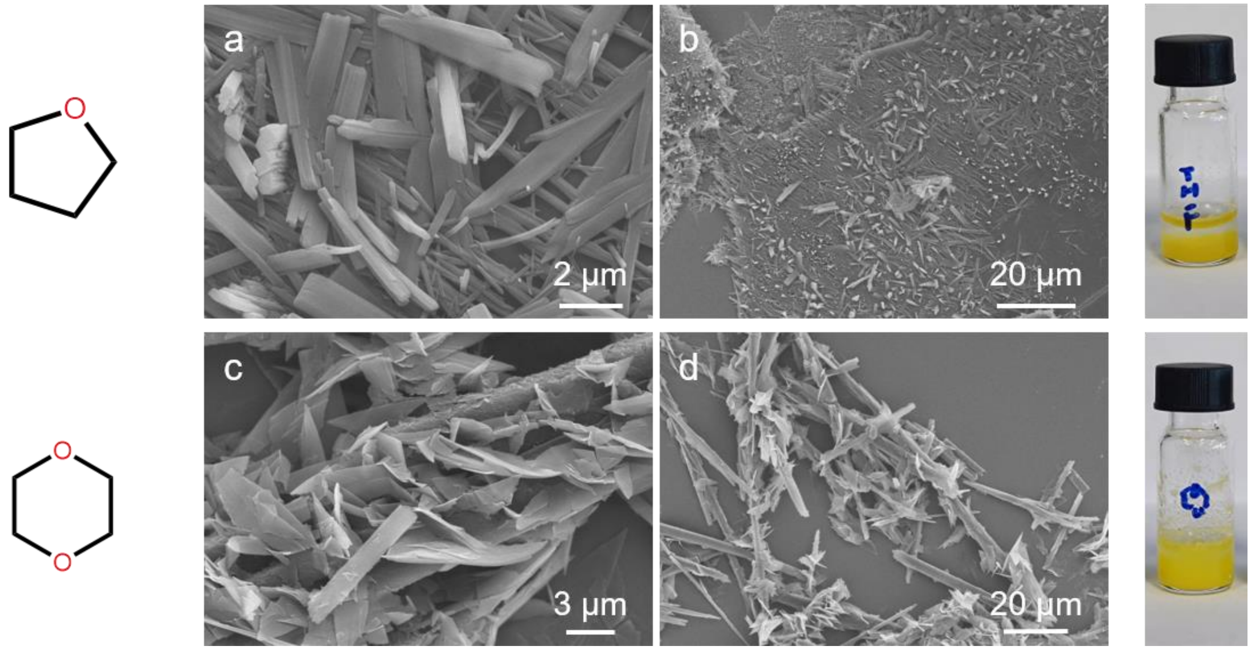

Supplementary Figure 11. Molecular structures and SEM images of *R*-Δ/Pyr co-assemblies obtained in different mixed solvents after annealing. (**a** and **b**) THF/H_2O. (**c** and **d**) dioxane/H_2O. (*R*-PMDI-Δ 1.0 mg, the mole ratio of *R*-PMDI-Δ/Pyr is 1 to 2 and the solvent volume ratio of organic solvent to water is 200 μL to 200 μL). PMDI-Δ and Pyr co-assembled in THF/H_2O or dioxane/H_2O to form a yellow suspension, indicating the formation of CT complexes between *R*-PMDI-Δ and Pyr. However, SEM characterization showed that no chiral structures can be observed.

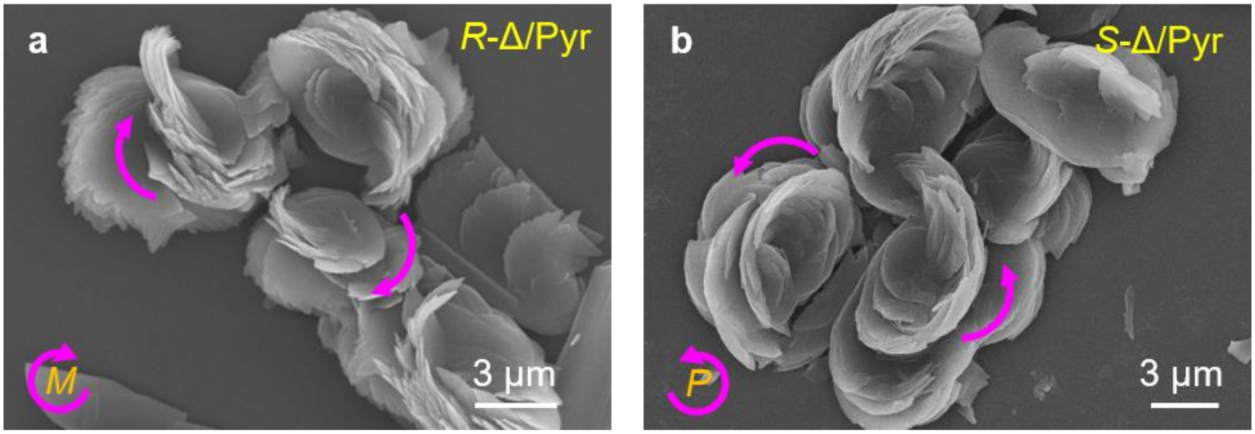

Supplementary Figure 12. SEM images of *R*-PMDI-Δ/Pyr helicoids^{NMP} (**a)** and *S*-PMDI-Δ/Pyr helicoids^{NMP} (**b**) obtained in NMP/H_2O after annealing.

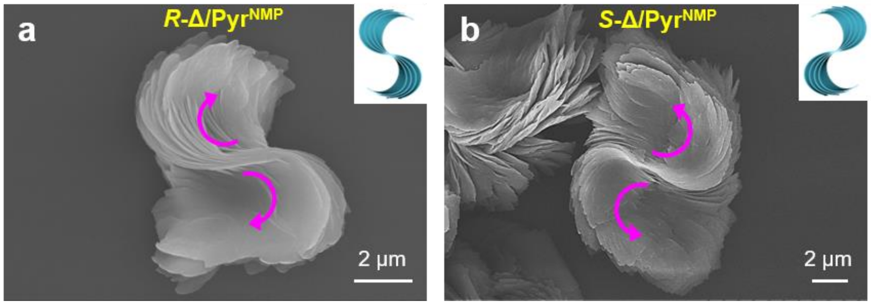

Supplementary Figure 13. SEM images of a fully-formed S-type double helicoid (for *R*-PMDI-Δ/Pyr) (**a**) and a fully-formed anti-S-shaped double helicoid (for *S*-PMDI-Δ/Pyr) (**b**) obtained in NMP/H_2O after annealing.

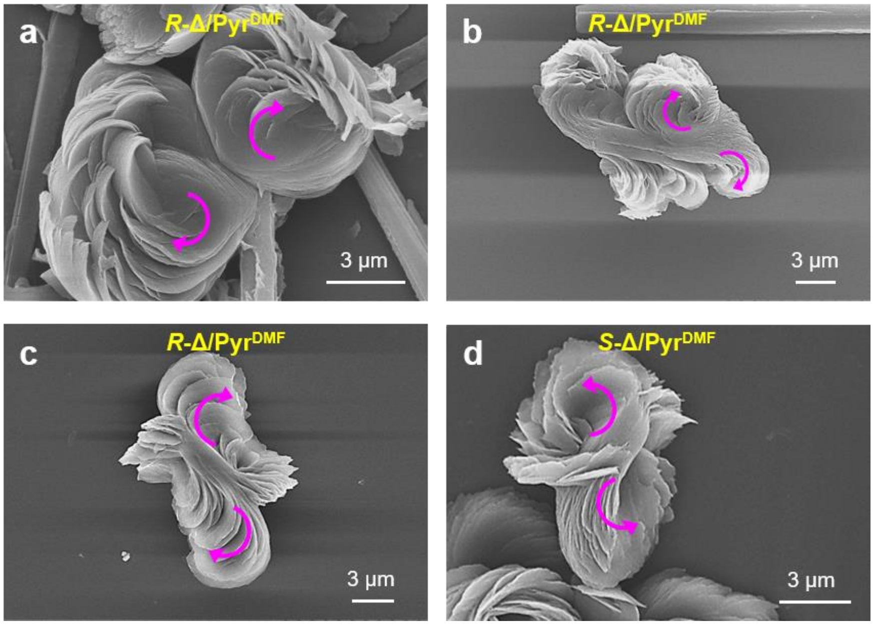

Supplementary Figure 14. (**a-d**) SEM images of microstructures between helicoids and double helicoids obtained in DMF/H_2O after annealing. These structures are formed by two helicoids side by side, resembling a double helicoid, but not completely connected and fully formed a whole.

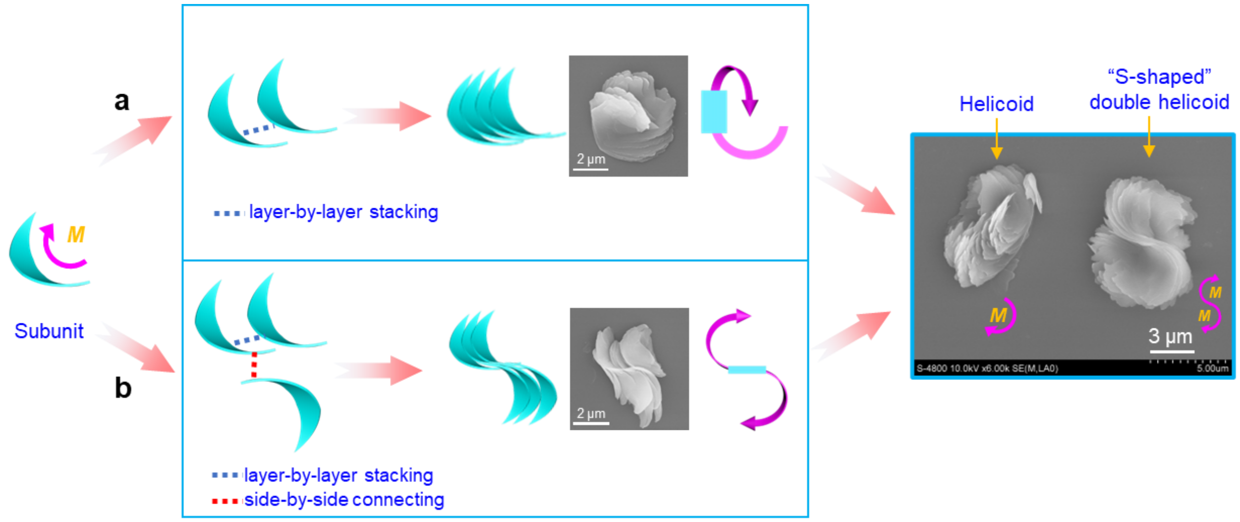

Supplementary Figure 15. Diagram of the formation mechanism of (**a**) helicoids and (**b**) double helicoids. The SEM images of helicoids and double helicoids in this figure were obtained by annealing assembly in NMP/H_2O mixed solvent.

Supplementary Table 2.

The viscosity and polarity parameters of solvents (H_2O, DMF and NMP)

Solvent	Viscosity (mPa·s)	Reichardt scale of polarity E_T^N
H_2O	1.00	1.000
DMF	0.92	0.386
NMP	1.67	0.355

The data in this table are derived from supplementary references (2-5).

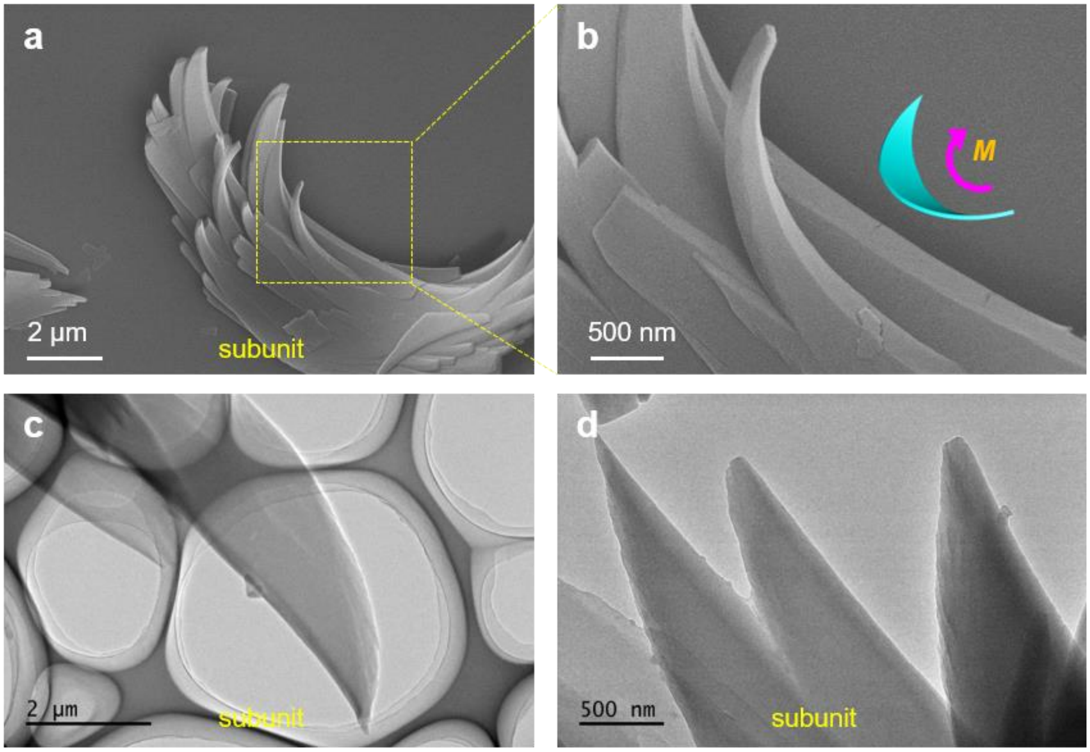

Supplementary Figure 16. (**a** and **b**) SEM and (**c** and **d**) TEM images of curved subunits of *R*-PMDI-Δ/Pyr helicoids^{DMF} and their corresponding cartoon representations. These subunits were formed by helical growth around screw dislocations.

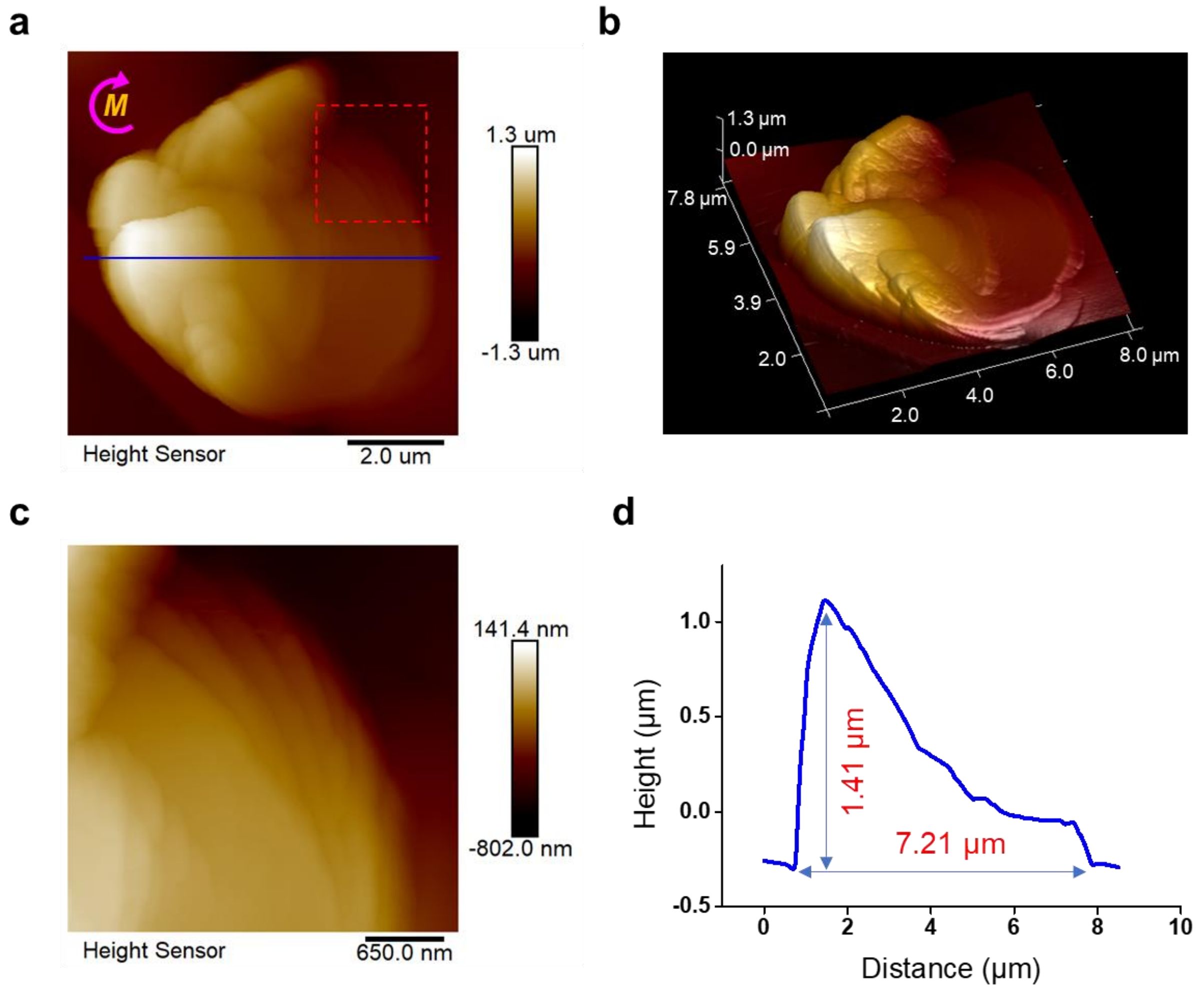

Supplementary Figure 17. AFM data of *R*-Δ/Pyr helicoid^{DMF} (**a**) AFM topography image; (**b**) three-dimensional (3D) AFM topography image. AFM images (**a**, **b**) clearly displaying the formation of an *M*-helical microstructure; (**c**) The AFM morphology image of the helicoid^{DMF} edge, as denoted by the red box in (**a**). (**d**) AFM height profile, as denoted by the blue line in (**a**), indicating a maximum height of ca. 1.41 μm and a maximum width of ca. 7.21 μm.

The 3D AFM morphology image and height profile of (**c**) were shown in Fig. 2 of the main text, corresponding to (**j**) and (**k**) respectively.

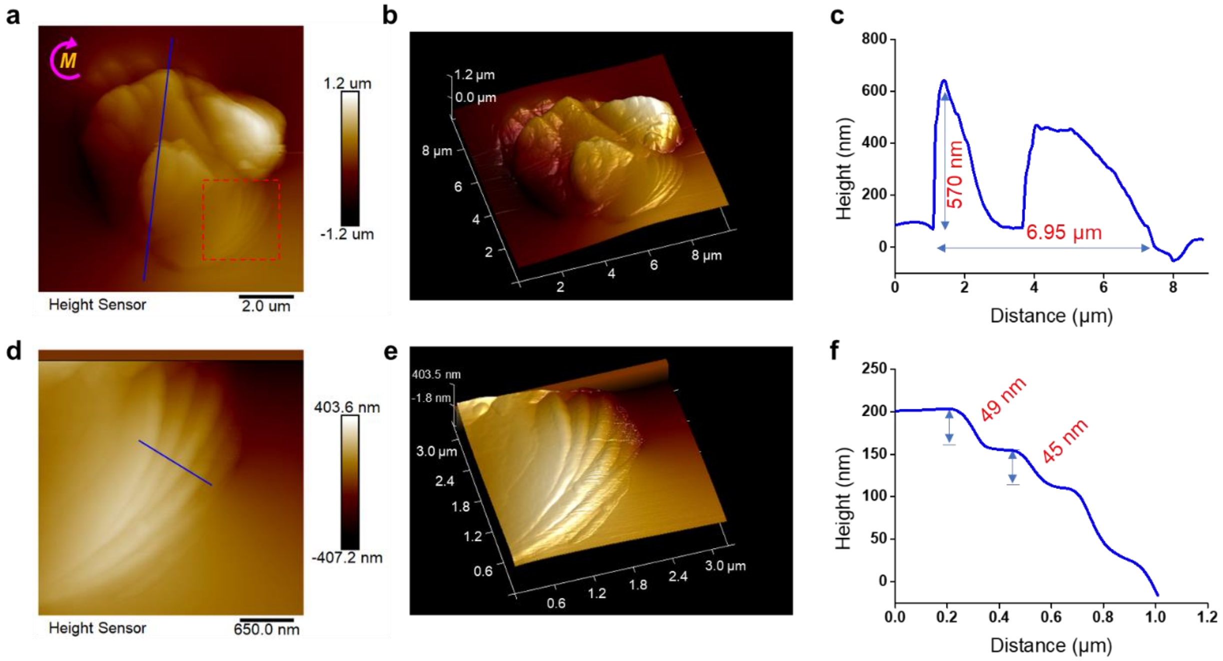

Supplementary Figure 18. AFM data of *R*-Δ/Pyr helicoid^{NMP} (**a**) AFM topography image; (**b**) three-dimensional (3D) AFM topography image. AFM images (**a**, **b**) clearly displaying the formation of an *M*-helical microstructure; (**c**) AFM height profile, as denoted by the blue line in (**a**), indicating a height of ca. 570 nm and a width of ca. 6.95 µm. (**d** and **e**) The AFM morphology image and 3D AFM morphology image of the red box in (**a**). (**f**) shows the height profile across the blue line in (**d**), indicating the heights of the subunit nanosheets are ca. 45-49 nm.

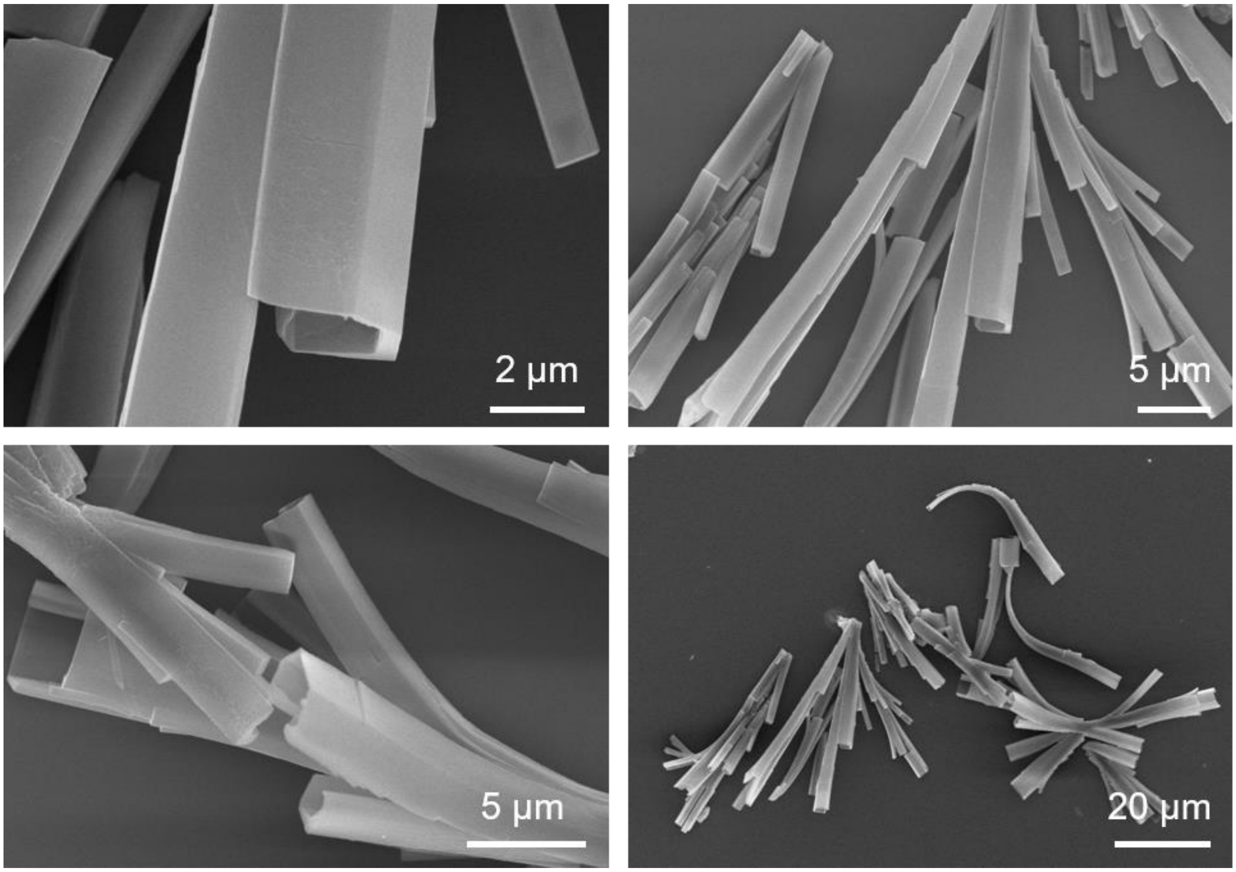

Supplementary Figure 19. SEM images of the *R*-PMDI-Δ/Pyr co-assemblies^{DMF} obtained before annealing.

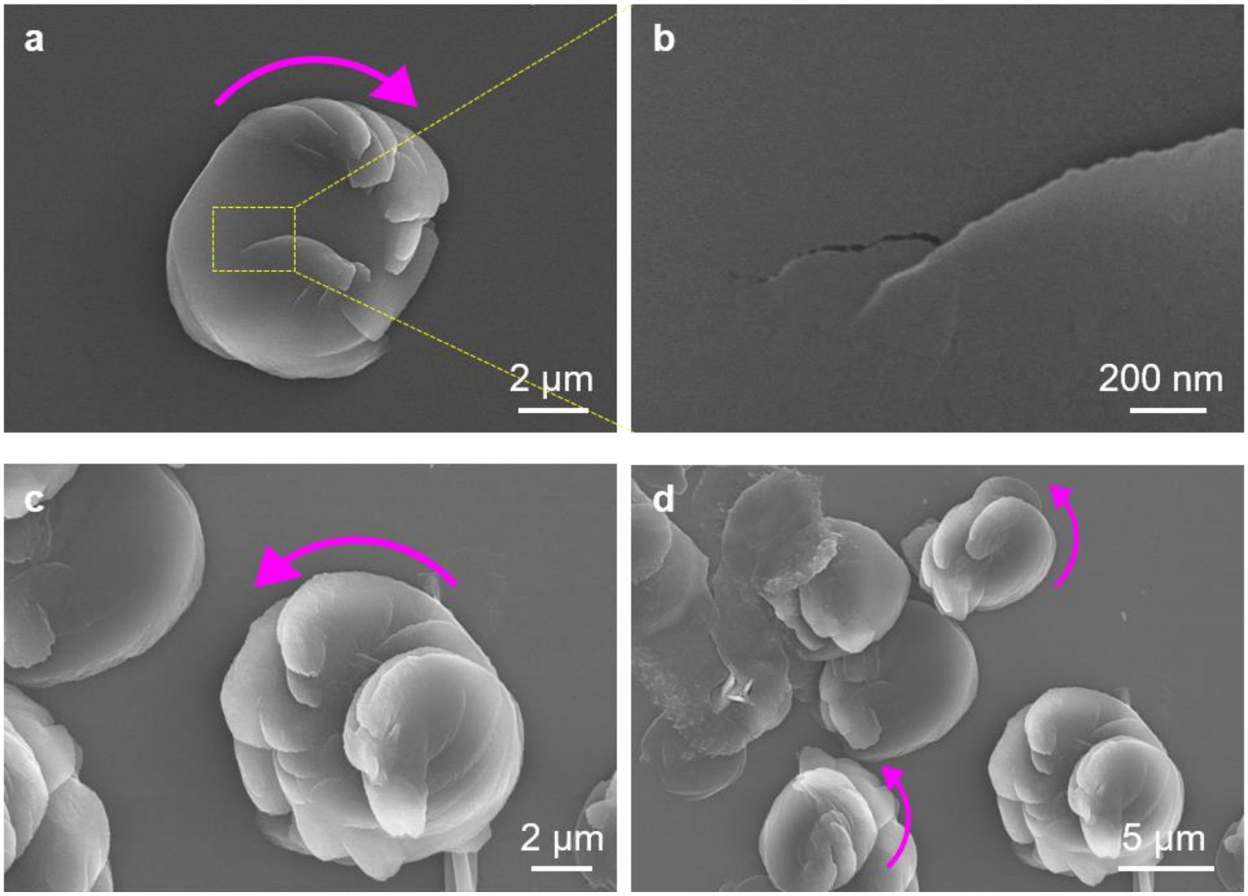

Supplementary Figure 20. SEM images of *R*-PMDI-Δ/Pyr helicoids^{NMP} (**a** and **b**) and *S*-PMDI-Δ/Pyr helicoids^{NMP} (**c** and **d**) obtained by natural cooling 10 minutes after annealing. We succeeded in capturing a few structures during the self-assembly process, which displayed loops present at their circular base. These features are consistent with spiral growth around a screw dislocation.

5. Supplementary single crystal data

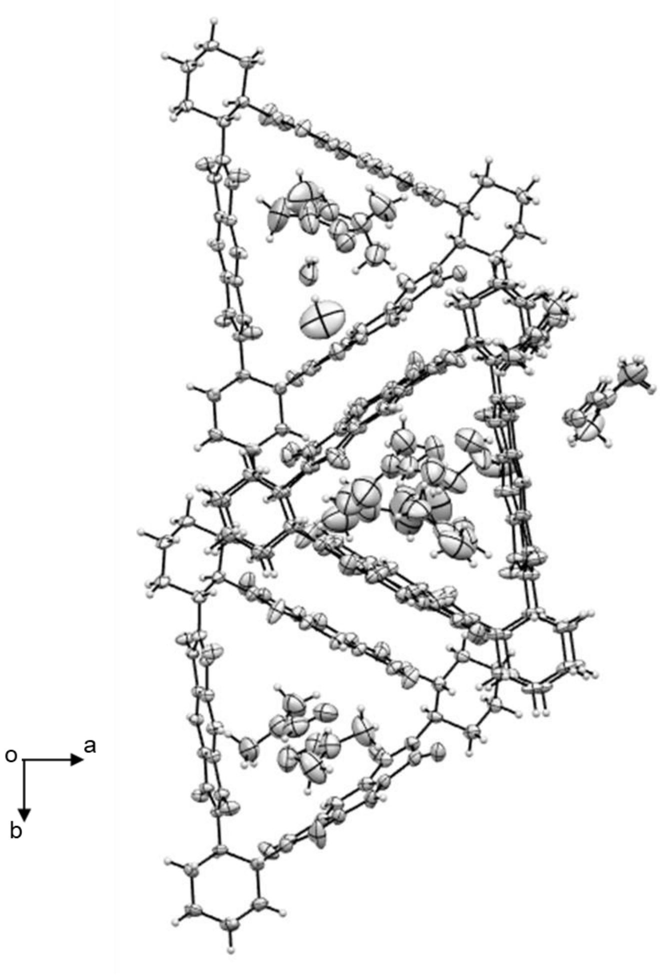

Supplementary Figure 21. An ORTEP diagram of *R*-PMDI-Δ single crystal^{DMF}, showing 50% probability ellipsoids. The CCDC number of *R*-PMDI-Δ single crystal^{DMF} is 2322342.

The following is a note on CheckCif file B-level alerts:

PLAT097_ALERT_2_B Large Reported Max. (Positive) Residual Density 1.57 eA-3

PLAT420_ALERT_2_B D-H Bond Without Acceptor O319 --H31G . Please Check

Author Response: This OH (O319-H31G) group is in a solvent H_2O molecule. Owing to a bit disorder of solvent molecules that could not be modeled adequately. These alerts do not affect the main results in this manuscript.

Supplementary Table 3.

Crystal data and structure refinement for *R*-PMDI-Δ single crystal[DMF].

Identification code	*R*-PMDI-Δ single crystal[DMF]
CCDC	2322342
Empirical formula	$C_{228}H_{238}N_{36}O_{65}$
Formula weight	4522.53
Temperature/K	169.99(10)
Crystal system	triclinic
Space group	P1
a/Å	15.9098(2)
b/Å	19.6507(3)
c/Å	21.2650(3)
α/°	65.3327(14)
β/°	68.5145(14)
γ/°	82.9549(13)
Volume/Å^3	5618.18(17)
Z	1
ρ_{calc}g/cm^3	1.337
μ/mm^{-1}	0.830
F(000)	2378.0
Crystal size/mm^3	0.2 × 0.15 × 0.15
Radiation	Cu Kα (λ = 1.54184)
2Θ range for data collection/°	4.88 to 154.104
Index ranges	-20 ≤ h ≤ 20, -24 ≤ k ≤ 23, -26 ≤ l ≤ 25
Reflections collected	194589
Independent reflections	42197 [R_{int} = 0.0288, R_{sigma} = 0.0187]
Data/restraints/parameters	42197/168/2979
Goodness-of-fit on F^2	1.966
Final R indexes [I>=2σ (I)]	R_1 = 0.0801, wR_2 = 0.2216
Final R indexes [all data]	R_1 = 0.0819, wR_2 = 0.2235
Largest diff. peak/hole / e Å^{-3}	1.57/-0.69
Flack parameter	0.02(2)

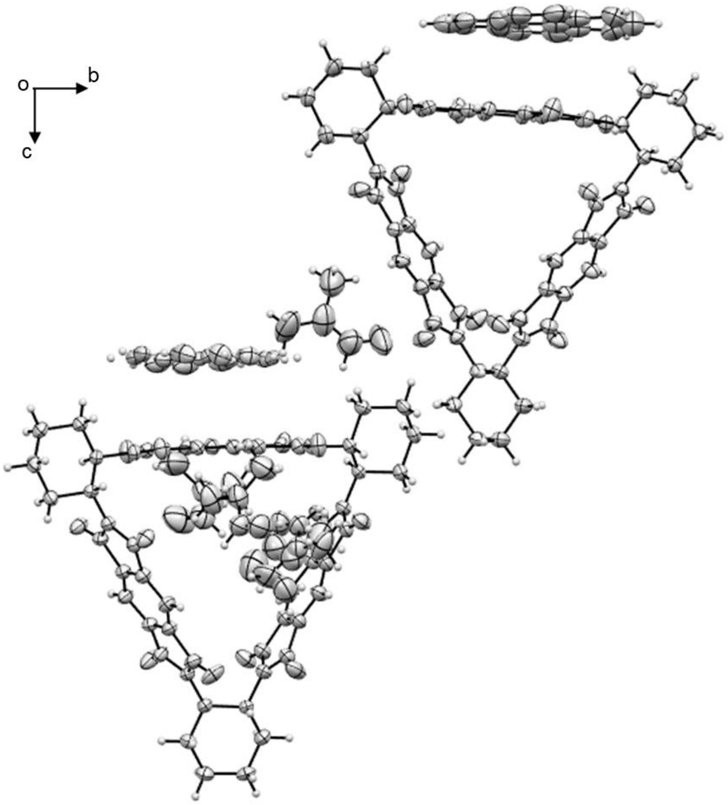

Supplementary Figure 22. An ORTEP diagram of *R*-PMDI-Δ/Pyr cocrystal^{DMF}, showing 50% probability ellipsoids. The CCDC number of *R*-PMDI-Δ/Pyr cocrystal^{DMF} is 2322343.

The following is a note on CheckCif file B-level alerts:

PLAT097_ALERT_2_B Large Reported Max. (Positive) Residual Density 1.36 eA-3

PLAT230_ALERT_2_B Hirshfeld Test Diff for C60 --C61 . 7.7 s.u.

PLAT230_ALERT_2_B Hirshfeld Test Diff for C62 --C63 . 8.7 s.u.

PLAT230_ALERT_2_B Hirshfeld Test Diff for C64 --C67_a . 7.3 s.u.

PLAT230_ALERT_2_B Hirshfeld Test Diff for C65 --C66 . 8.9 s.u.

PLAT230_ALERT_2_B Hirshfeld Test Diff for C67 --C64_a . 7.3 s.u.

Author Response: The best quality crystal has been mounted. Owing to a bit disorder in the rings that could not be modeled adequately. We are confident that this is a pyrene.

Supplementary Table 4.

Crystal data and structure refinement for *R*-PMDI-Δ/Pyr cocrystal^{DMF}.

Identification code	*R*-PMDI-Δ/Pyr cocrystal^{DMF}
CCDC	2322343
Empirical formula	$C_{124}H_{110}N_{16}O_{28}$
Formula weight	2272.27
Temperature/K	170.00(15)
Crystal system	monoclinic
Space group	C2
a/Å	36.5878(7)
b/Å	13.61180(10)
c/Å	32.2822(6)
α/°	90
β/°	130.317(3)
γ/°	90
Volume/Å^3	12258.6(5)
Z	4
ρ_{calc}g/cm^3	1.231
μ/mm^{-1}	0.734
F(000)	4760.0
Crystal size/mm^3	0.3 × 0.1 × 0.05
Radiation	Cu Kα (λ = 1.54184)
2Θ range for data collection/°	3.59 to 154.238
Index ranges	-46 ≤ h ≤ 45, -16 ≤ k ≤ 17, -40 ≤ l ≤ 40
Reflections collected	82515
Independent reflections	24432 [R_{int} = 0.0392, R_{sigma} = 0.0326]
Data/restraints/parameters	24432/530/1643
Goodness-of-fit on F^2	1.026
Final R indexes [I>=2σ (I)]	R_1 = 0.0600, wR_2 = 0.1718
Final R indexes [all data]	R_1 = 0.0664, wR_2 = 0.1790
Largest diff. peak/hole / e Å^{-3}	1.36/-0.47
Flack parameter	0.06(5)

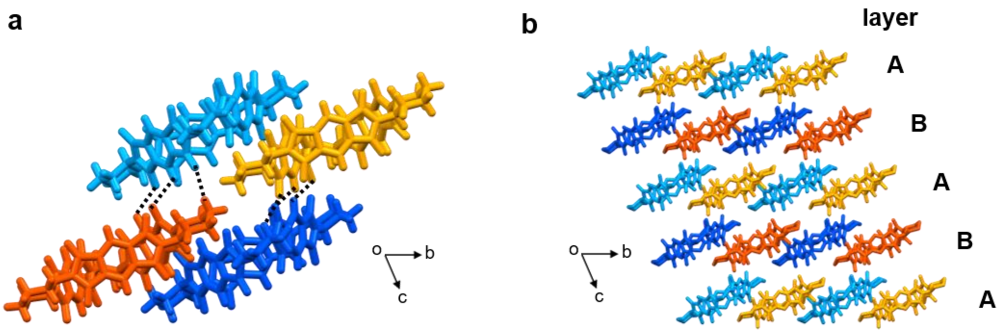

Supplementary Figure 23. The single crystal structure of *R*-Δ. (**a**) multiple C-H···O hydrogen bonds ($d_{(O\cdots H)}$ 2.5-2.7Å) between PMDI-Δ molecules of adjacent layers. (**b**) The layered structures (AB-stacking model) along the *c*-axis.

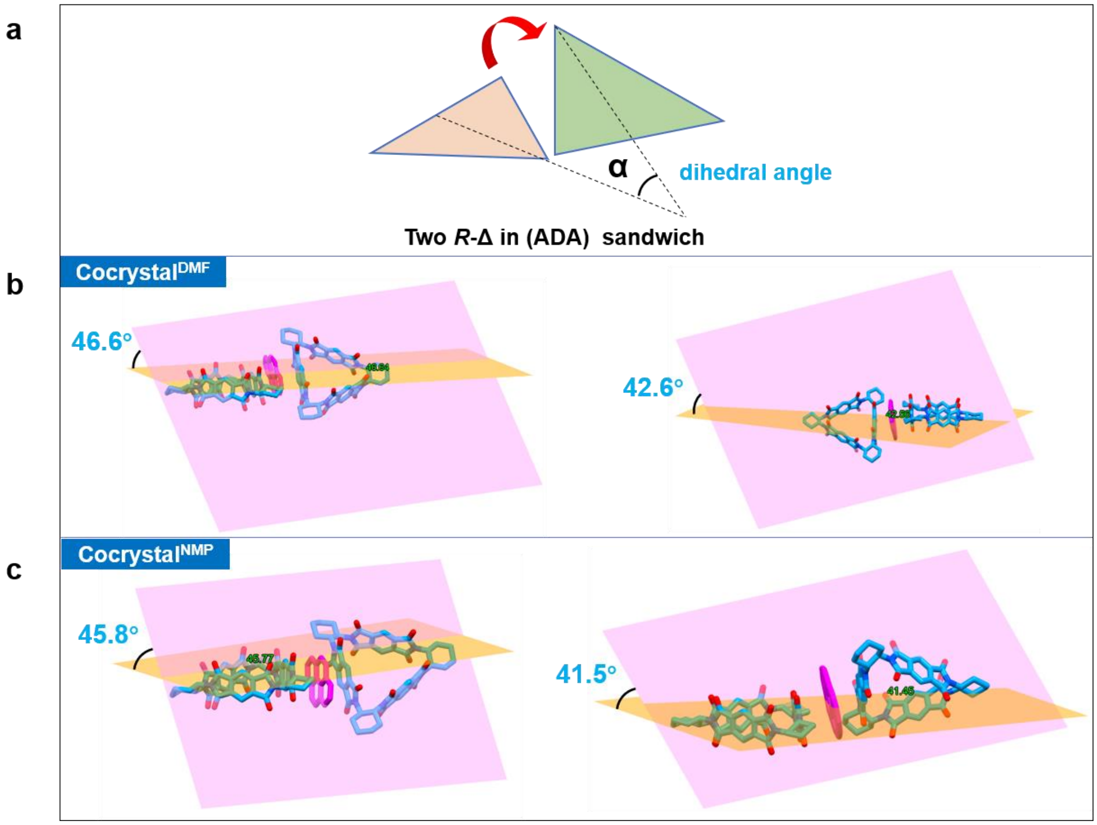

Supplementary Figure 24. (**a**) Diagram of dihedral angle α defined as the dihedral angle between two PMDI-Δ molecular planes in an ADA sandwich. The different colored parallelograms represent the molecular planes of two *R*-PMDI-Δ in an ADA sandwich. The dihedral angle α for cocrystal^{DMF} (**b**) and for cocrystal^{NMP} (**c**) are slightly different.

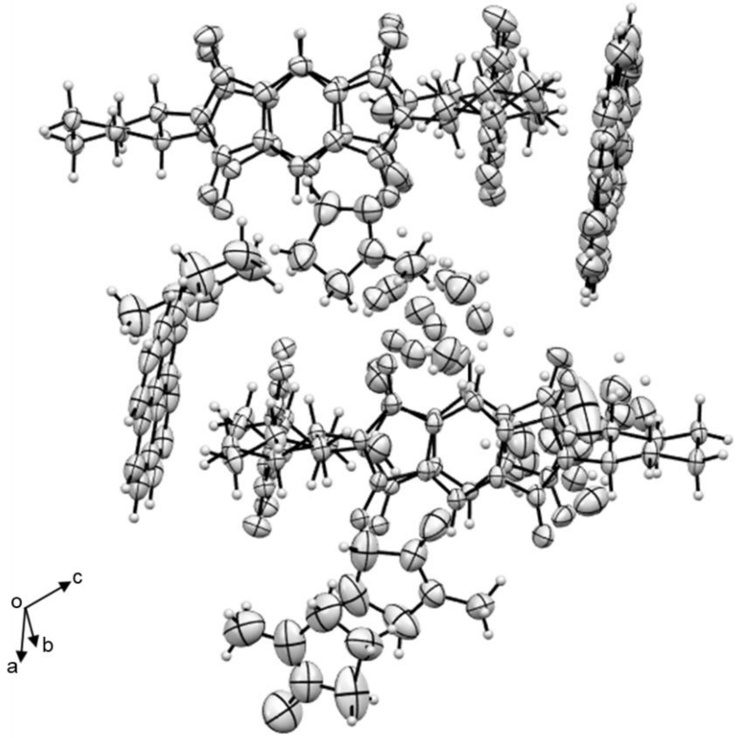

Supplementary Figure 25. An ORTEP diagram of *R*-PMDI-Δ/Pyr cocrystal^{NMP}, showing 50% probability ellipsoids. The CCDC number of *R*-PMDI-Δ/Pyr cocrystal^{NMP} is 2322344.

Supplementary Table 5.

Crystal data and structure refinement for *R*-PMDI-Δ/Pyr cocrystal^{NMP}.

Identification code	*R*-PMDI-Δ/Pyr cocrystal^{NMP}
CCDC	2322344
Empirical formula	$C_{142}H_{140.93}N_{18}O_{32.47}$
Formula weight	2619.09
Temperature/K	169.99(11)
Crystal system	monoclinic
Space group	C2
a/Å	36.7894(3)
b/Å	13.86140(10)
c/Å	29.4292(2)
α/°	90
β/°	122.2530(10)
γ/°	90
Volume/Å^3	12691.8(2)
Z	4
ρ_{calc}g/cm^3	1.371
μ/mm^{-1}	0.814
F(000)	5515.0
Crystal size/mm^3	0.1 × 0.1 × 0.01
Radiation	Cu Kα (λ = 1.54178)
2Θ range for data collection/°	4.832 to 153.786
Index ranges	$-43 \leq h \leq 46$, $-15 \leq k \leq 17$, $-36 \leq l \leq 36$
Reflections collected	124250
Independent reflections	24541 [R_{int} = 0.0343, R_{sigma} = 0.0260]
Data/restraints/parameters	24541/740/1948
Goodness-of-fit on F^2	1.029
Final R indexes [I>=2σ (I)]	R_1 = 0.0528, wR_2 = 0.1463
Final R indexes [all data]	R_1 = 0.0563, wR_2 = 0.1494
Largest diff. peak/hole / e Å^{-3}	0.76/-0.42
Flack parameter	0.06(4)

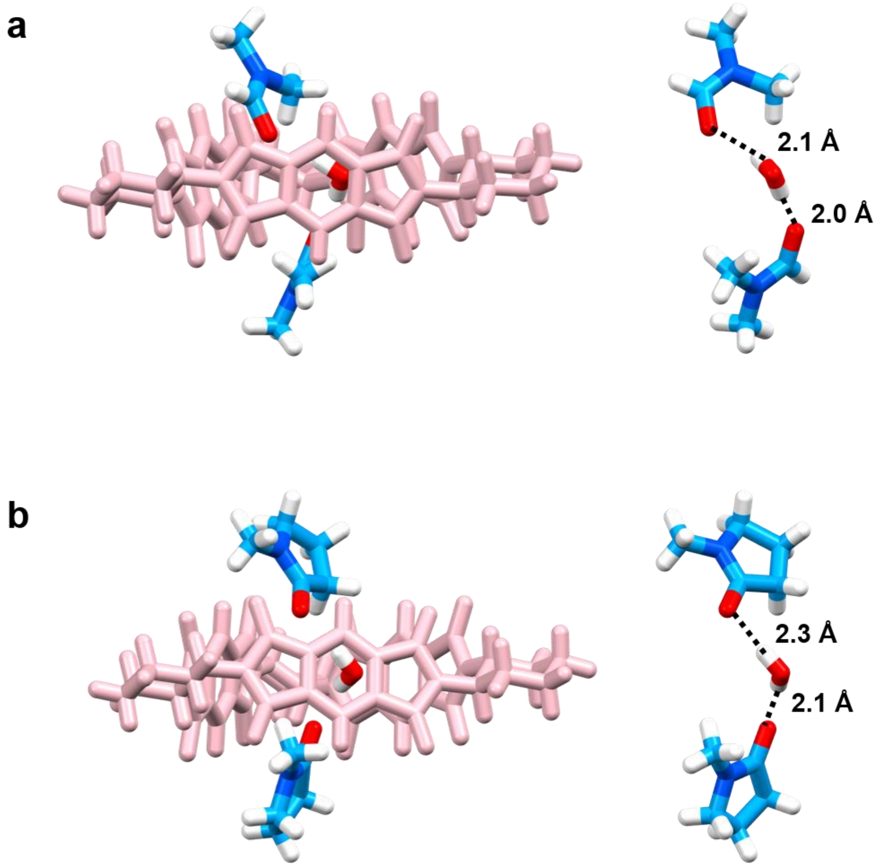

Supplementary Figure 26. The hydrogen bond between (**a**) DMF or (**b**) NMP and H_2O in the intrinsic pore of *R*-PMDI-Δ. The structures (**a**) and (**b**) were extracted from *R*-PMDI-Δ single crystal^{DMF} and *R*-PMDI-Δ/Pyr cocrystal^{NMP}, respectively.

6. Supplementary density functional theory calculations

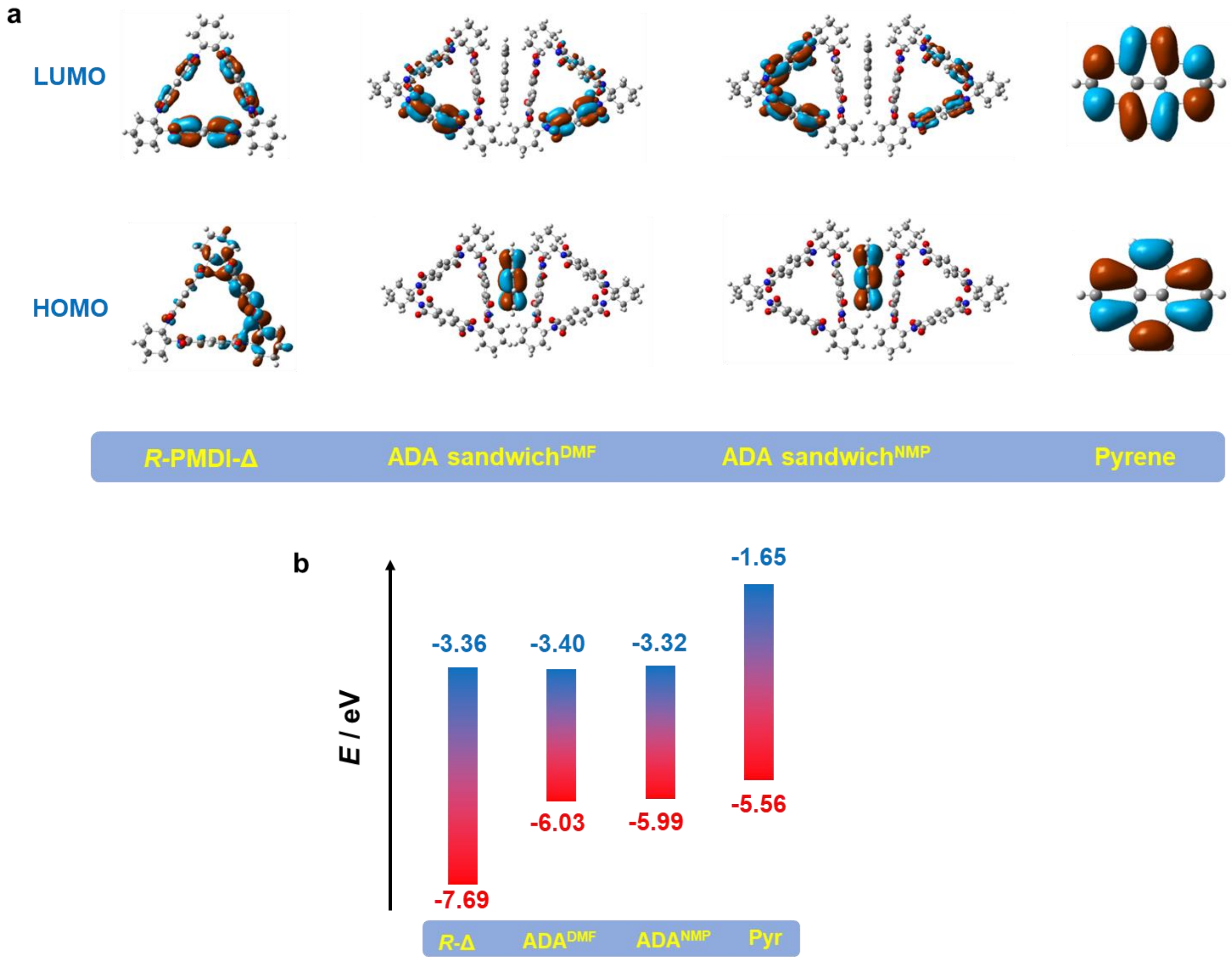

Supplementary Figure 27. (**a** and **b**) Frontier molecular orbitals and energy level diagrams of *R*-PMDI-Δ, ADA sandwich^{DMF}, ADA sandwich^{NMP} and Pyr calculated by DFT at Gaussian 09 program at B3LYP 6-311G** level [6].

7. Supplementary spectra

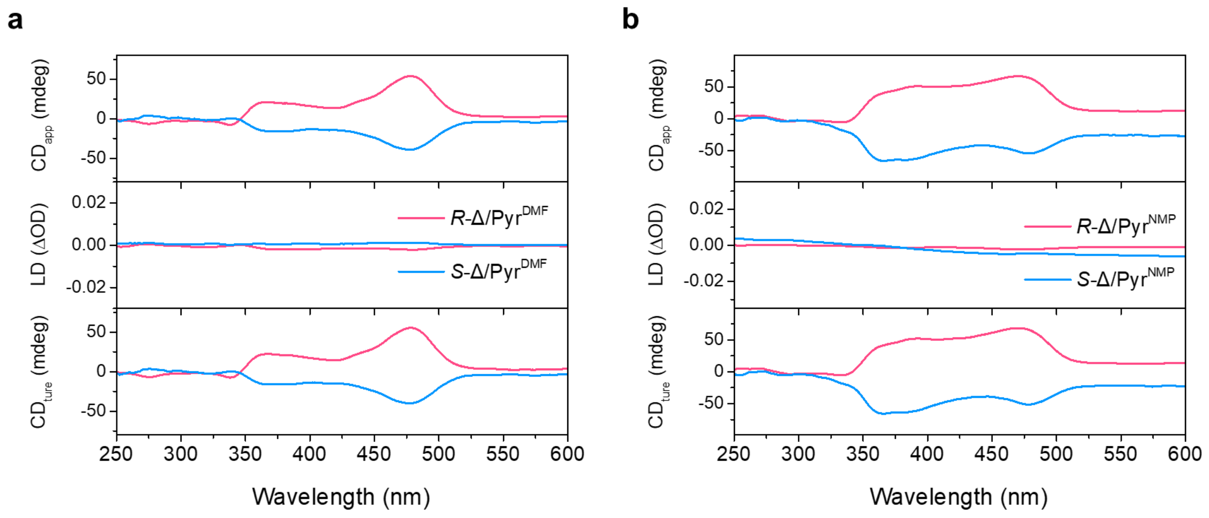

Supplementary Figure 28. The apparent CD (CD_{app}, upper), LD (middle) and true CD (CD_{true}, bottom) signals of PMDI-Δ/Pyr helicoids obtained in (**a**) DMF/H_2O and (**b**) NMP/H_2O. In order to evaluate the contribution of LD to CD, LD and CD spectra have been simultaneously measured [7-9]. The true CD signals (CD_{true}) can be calculated by the following semi-empirical equation according to the previous literature [7,10], $CD_{true} = CD_{app} - LD \times 0.02$. It was found that the contribution of LD to CD of all the assemblies was negligible in the present experiment.

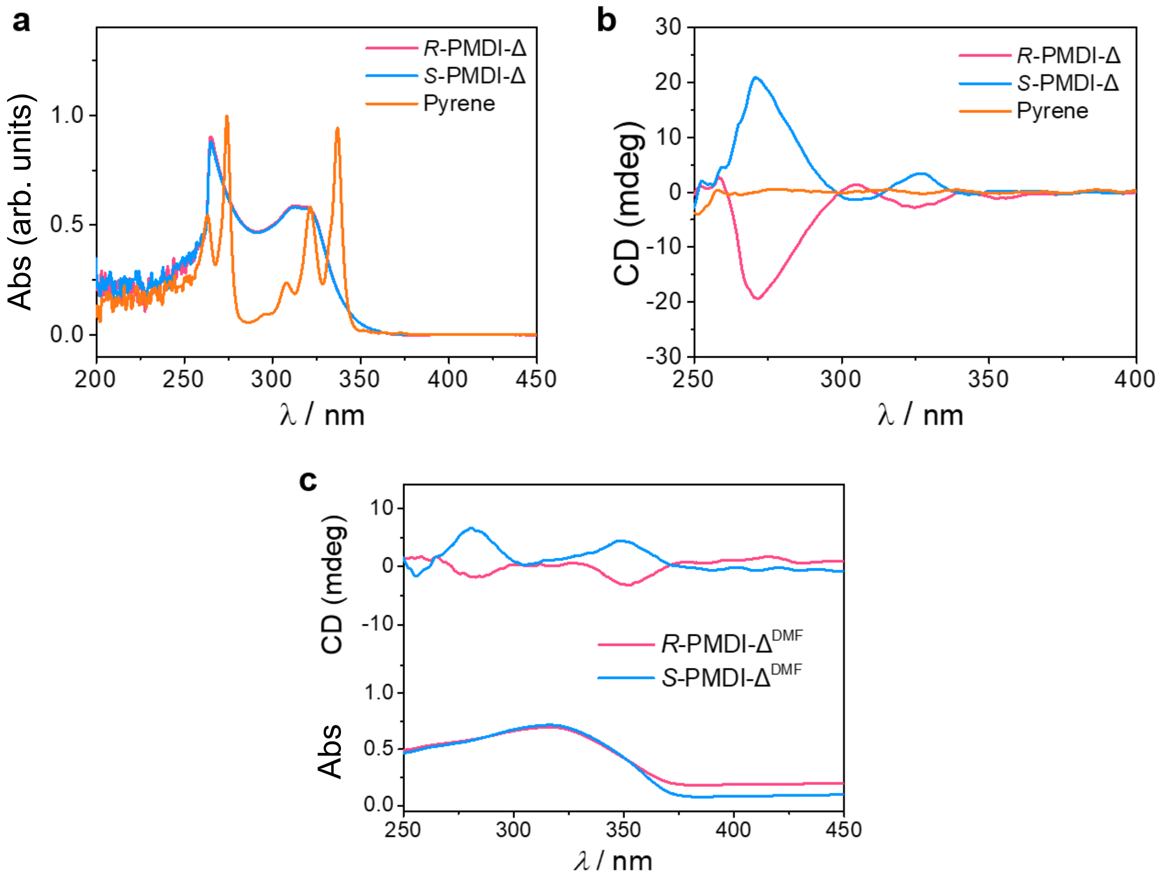

Supplementary Figure 29. (**a**) UV-Vis and (**b**) CD spectra of *R*-PMDI-Δ, *S*-PMDI-Δ and Pyrene in DMF. [*R*-PMDI-Δ] = [*S*-PMDI-Δ] = [Pyrene] = 0.1 mM. (**c**) CD spectra of PMDI-Δ self-assemblies^{DMF}.

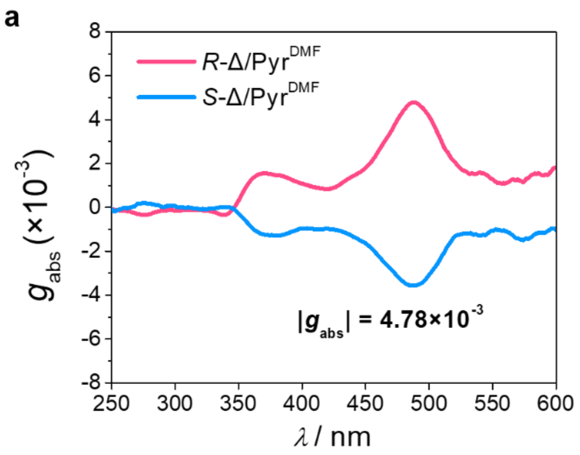

Supplementary Figure 30. Absorptive dissymmetry factor (g_{abs}) spectra of PMDI-Δ/Pyr co-assemblies obtained in (**a**) DMF/H_2O and (**b**) NMP/H_2O, respectively.

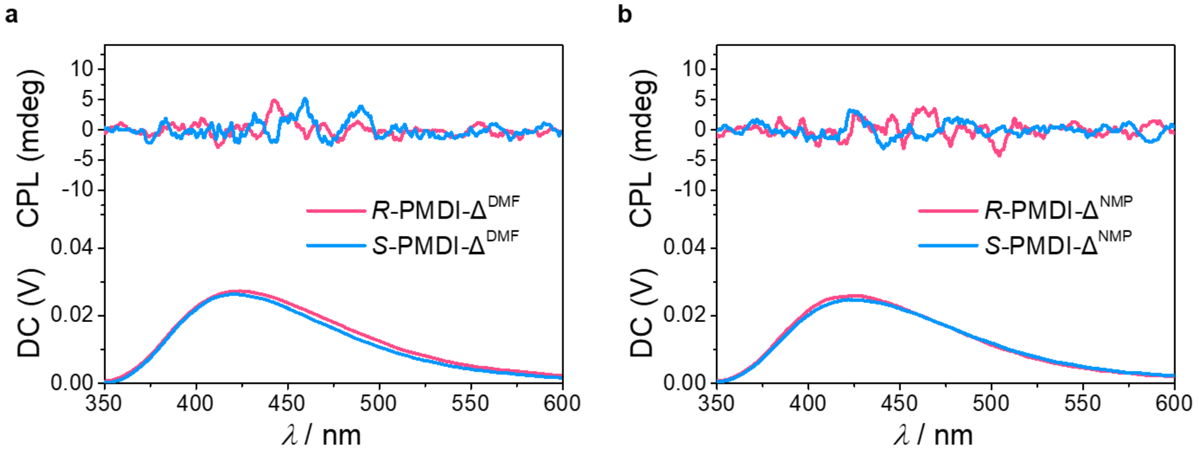

Supplementary Figure 31. CPL spectra of PMDI-Δ self-assemblies obtained in (**a**) DMF/H_2O and (**b**) NMP/H_2O, respectively. λ_{ex} = 300 nm for CPL measurements. No CPL signal can be detected. The low DC values were caused by the weak fluorescence properties of PMDI-Δ self-assemblies.

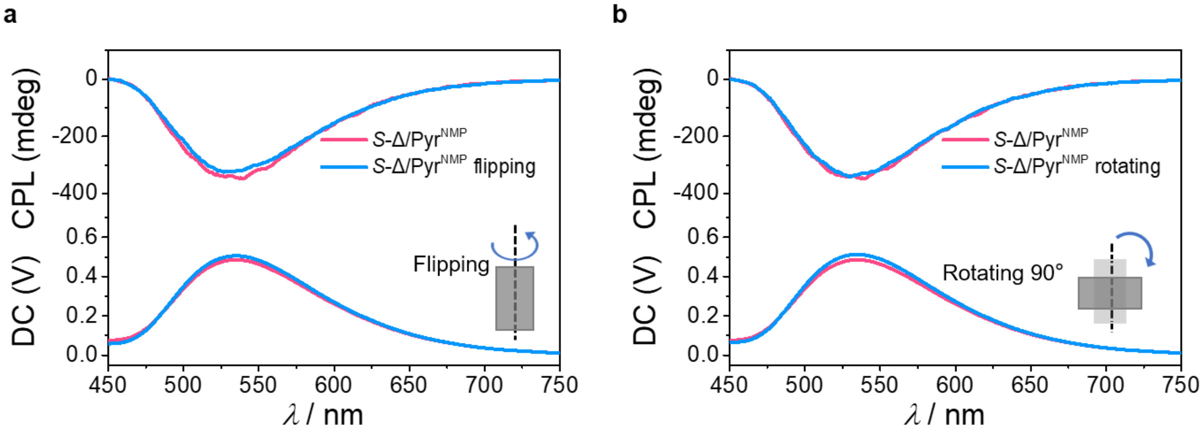

Supplementary Figure 32. The CPL spectra of *S*-PMDI-Δ/Pyr helicoids^{NMP} measured by (**a**) flipping and (**b**) rotating. the intensity of the CPL signal was almost the same when the sample was flipped or rotated 90°, indicating that the contribution of LD artifacts could be neglected in this system.

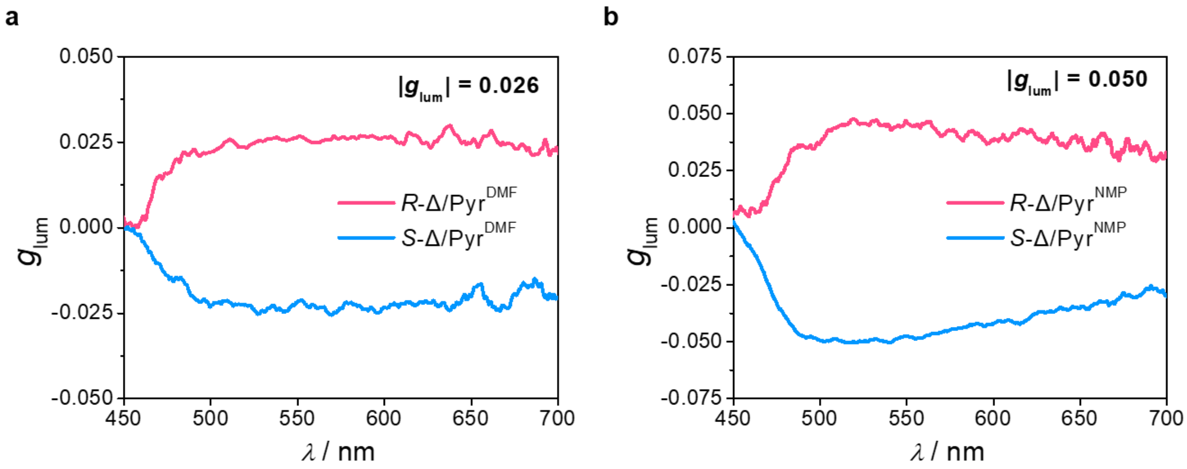

Supplementary Figure 33. Luminescent dissymmetry factor (g_{lum}) spectra of PMDI-Δ/Pyr co-assemblies obtained in (**a**) DMF/H_2O and (**b**) NMP/H_2O, respectively. λ_{ex} = 365 nm for CPL measurements.

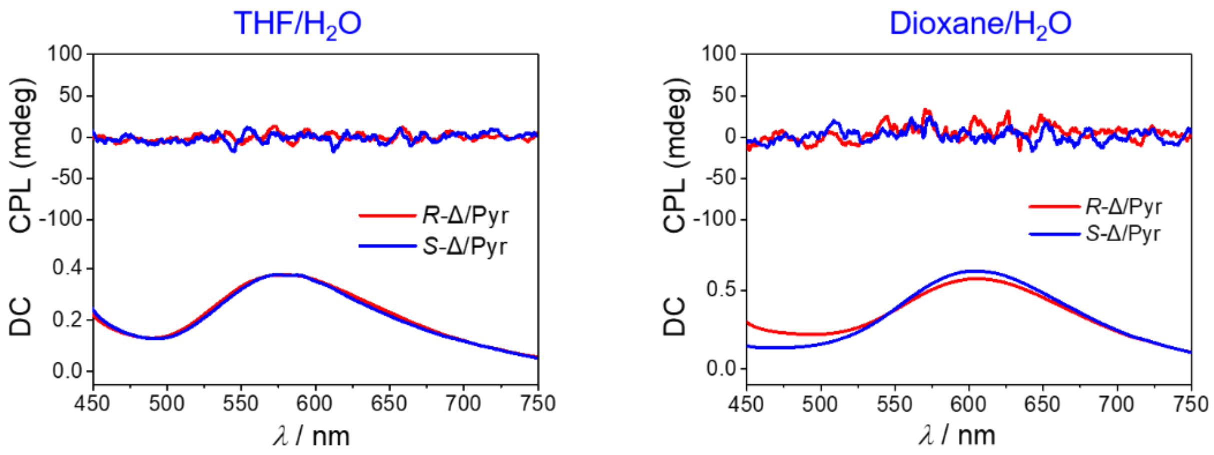

Supplementary Figure 34. CPL spectra of PMDI-Δ/Pyr co-assemblies obtained in THF/H_2O and dioxane/H_2O, respectively.

Supplementary Table 6.

Photophysical Data for Helicoids

	λ_{abs} /nm	λ_{em} /nm	$\Delta\tilde{v}_{Stokes}$ /nm	$\lvert g_{abs}\rvert$	$\lvert g_{lum}\rvert$ at λ_{em}	Φ_F (%)	τ_{avg} (ns)	K_{fl} [$10^6 s^{-1}$][a]	K_{nr} [$10^7 s^{-1}$][b]
**Helicoids^{DMF}**	**432**	**533**	**101**	**0.00478**	**0.02594**	**5.27**	**19.42**	**2.71**	**4.88**
**Helicoids^{NMP}**	**432**	**539**	**107**	**0.00584**	**0.05014**	**5.68**	**16.95**	**3.35**	**5.56**

[a] $K_{fl} = \Phi_F/\tau_{avg}$, where Φ_F and τ_{avg} are the fluorescence quantum yield and lifetime, respectively.
[b] $K_{nr} = 1/\tau_{avg} - K_{fl}$

8. Supplementary ^{1}H-NMR spectra of helicoids

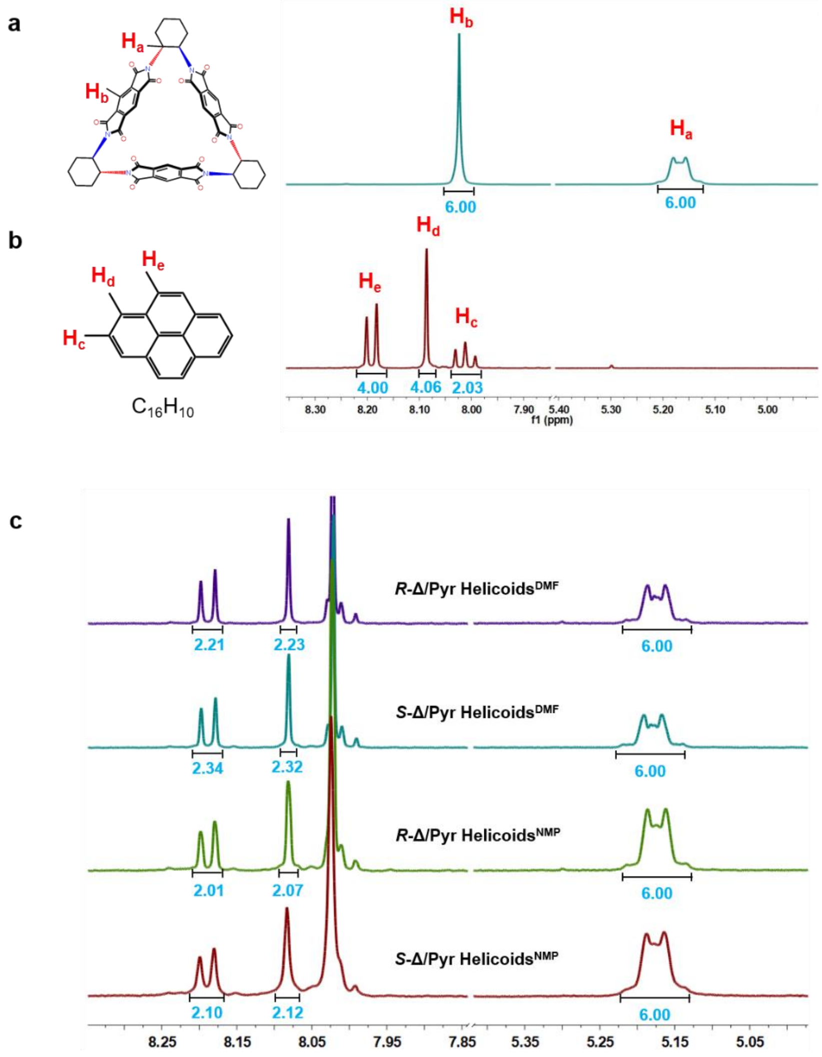

Supplementary Figure 35. (**a** and **b**) Molecular structures and partial ^{1}H-NMR spectra of *R*-PMDI-Δ and Pyr, respectively (400 MHz, $CDCl_3$, 298K). (**c**) Partial ^{1}H-NMR spectra of PMDI-Δ/Pyr helicoids (400 MHz, $CDCl_3$, 298K).

Supplementary Table 7.

The actual mole ratio of PMDI-Δ and Pyr in helicoids obtained by integral area of the ^{1}H-NMR spectra in Supplementary Figure 35c. The results indicated that the actual ratio of PMDI-Δ and Pyr in the helicoids obtained after annealing is close to 2:1, which is the same as that in cocrystals.

Coassemblies	^{1}H-NMR Integral ratio PMDI-Δ : Pyr	Approximate ratio PMDI-Δ : Pyr
R-Δ/Pyr Helicoids^{NMP}	1:0.51	≈ 2:1
S-Δ/Pyr Helicoids^{NMP}	1:0.53	≈ 2:1
R-Δ/Pyr Helicoids^{DMF}	1:0.56	≈ 2:1
S-Δ/Pyr Helicoids^{DMF}	1:0.58	≈ 2:1

9. Supplementary XRD patterns

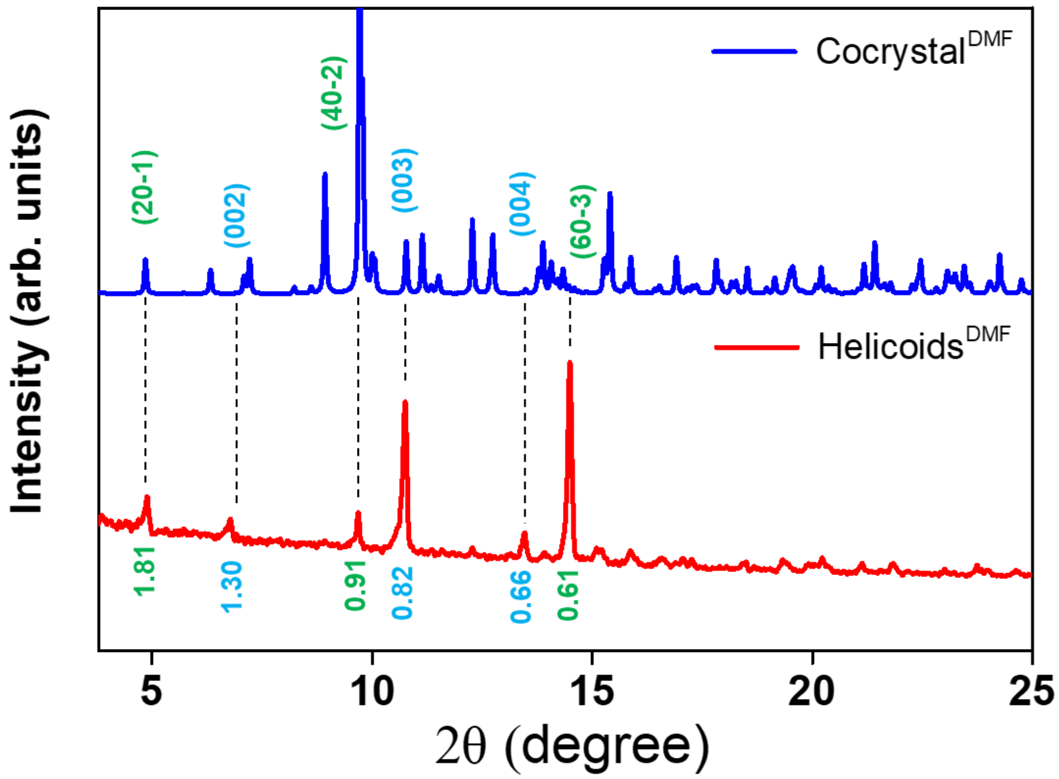

Supplementary Figure 36. Experimental XRD of helicoids^{DMF} and simulated XRD of cocrystal^{DMF}. The XRD pattern of the helicoids^{DMF} was consistent with the XRD pattern of cocrystal^{DMF} simulation.

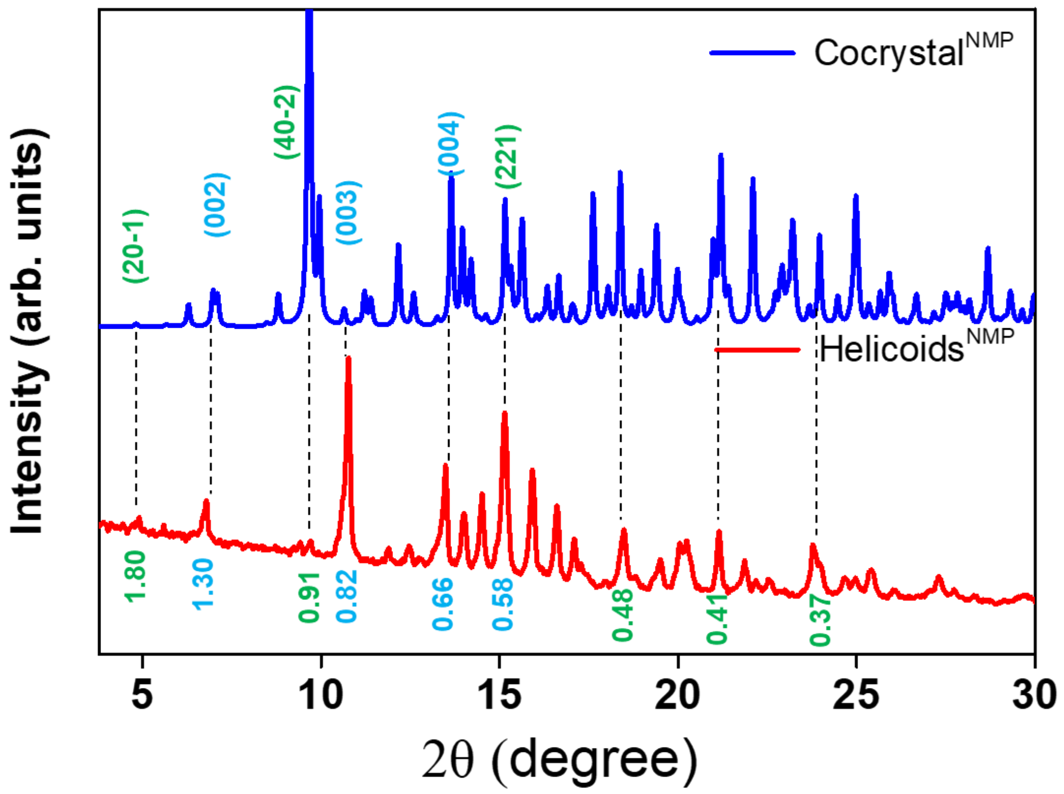

Supplementary Figure 37. Experimental XRD of helicoids^{NMP} and simulated XRD of cocrystal^{NMP}. The XRD pattern of the helicoids^{NMP} was consistent with the XRD pattern of cocrystal^{NMP} simulation.

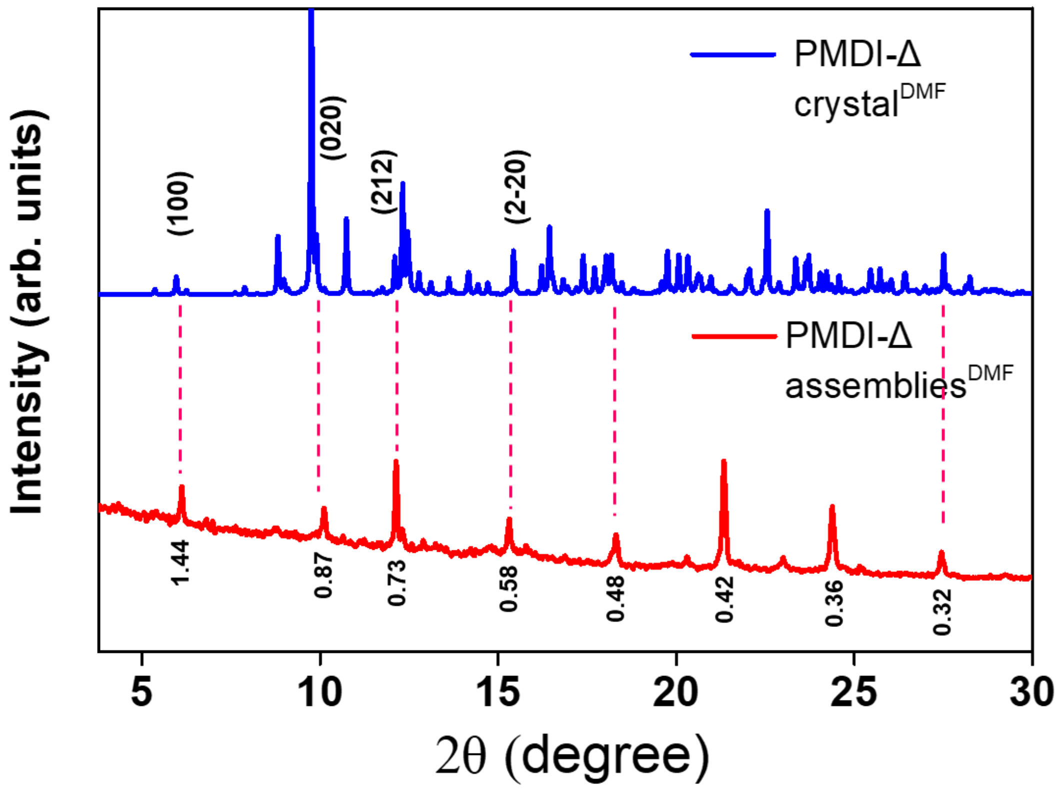

Supplementary Figure 38. Experimental XRD of PMDI-Δ assemblies^{DMF} and simulated XRD of PMDI-Δ crystal^{DMF}. The XRD pattern of the PMDI-Δ assemblies^{DMF} was consistent with the XRD pattern of PMDI-Δ crystal^{DMF} simulation.

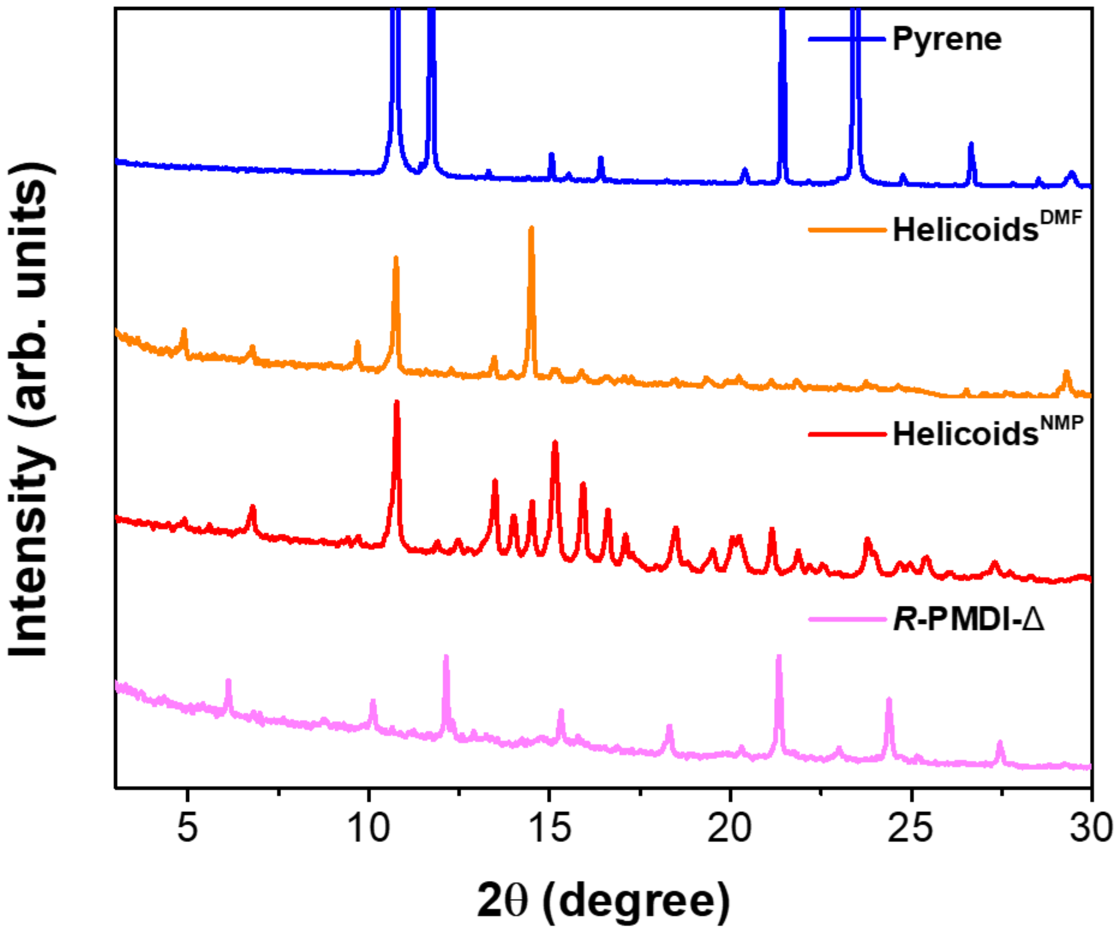

Supplementary Figure 39. Powder X-ray diffraction data of pyrene powder obtained in DMF/H_2O (blue) and, PMDI-Δ/Pyr helicoids^{DMF} (orange), PMDI-Δ/Pyr helicoids^{NMP} (red) and individual PMDI-Δ assemblies^{DMF} (magenta). In comparison with the powder X-ray diffraction patterns of individual PMDI-Δ and pyrene powders, those for the helicoids display new diffraction peaks, illustrating the formation of PMDI-Δ/Pyr co-assembiles.

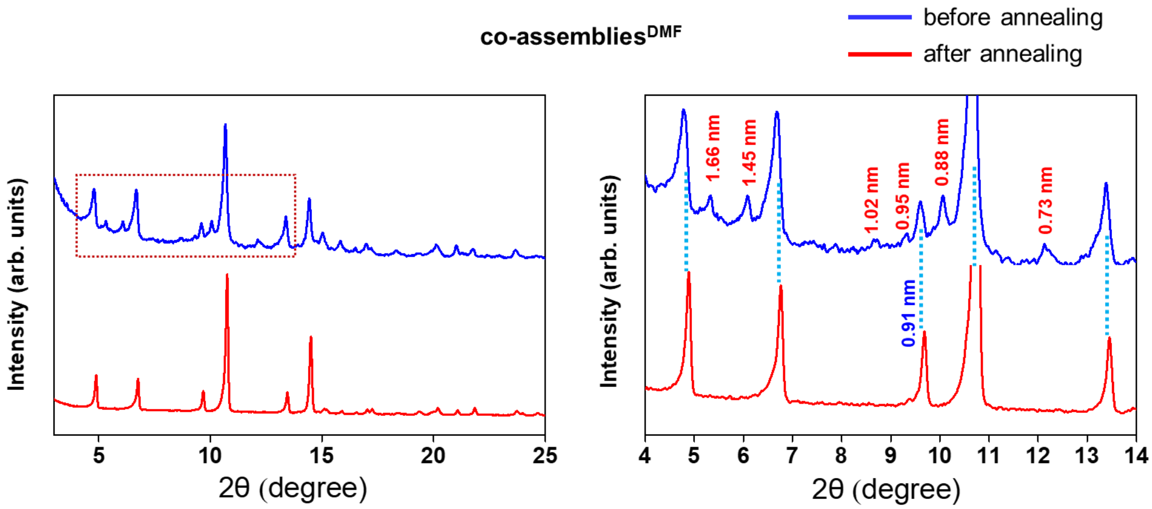

Supplementary Figure 40. Comparison of PXRD patterns of the co-assemblies^{DMF} before and after annealing. The figure on the right is an enlarged view of the selected part of the red dotted box in the left figure.

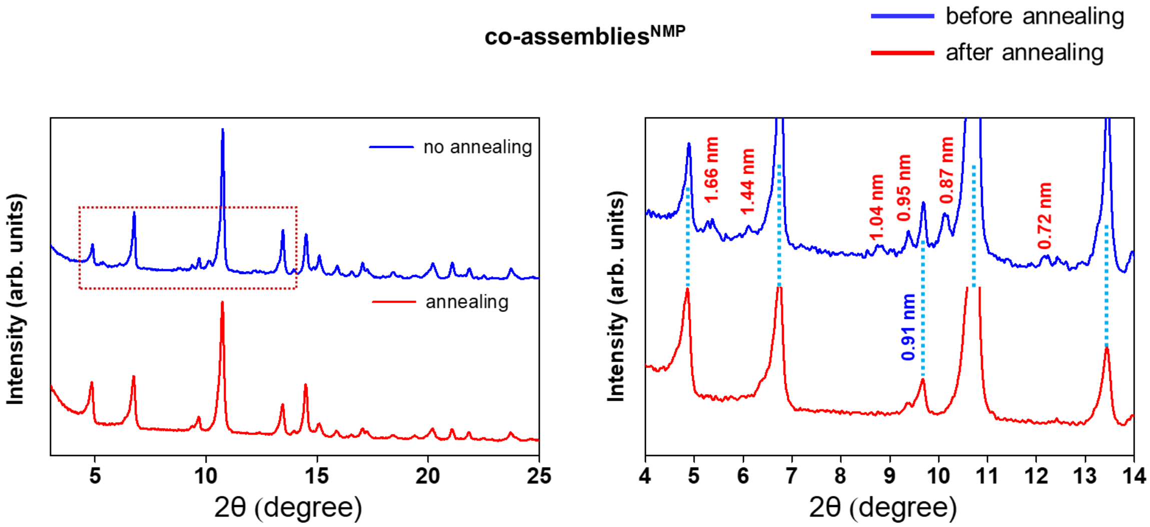

Supplementary Figure 41. Comparison of PXRD patterns of the co-assemblies^{NMP} before and after annealing. The figure on the right is an enlarged view of the selected part of the red dotted box in the left figure.

10. Supplementary NMR and MS spectra

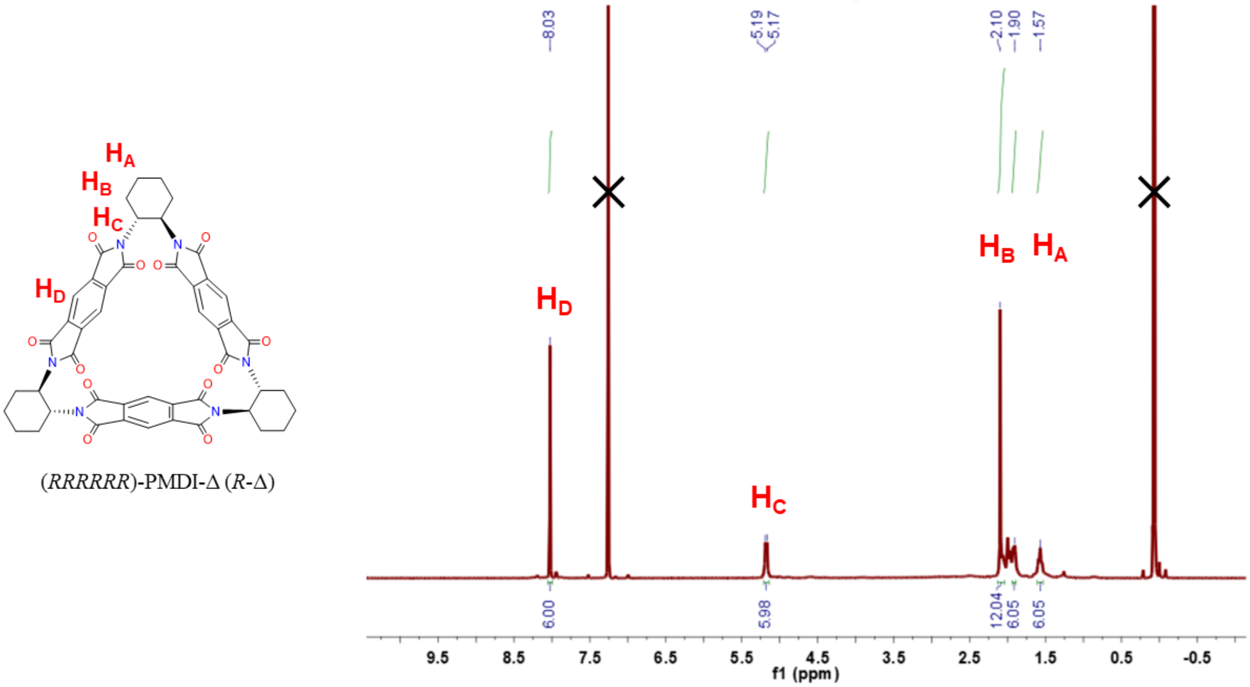

Supplementary Figure 42. ^{1}H-NMR spectrum of *R*-Δ (400 MHz, $CDCl_3$, 298 K).

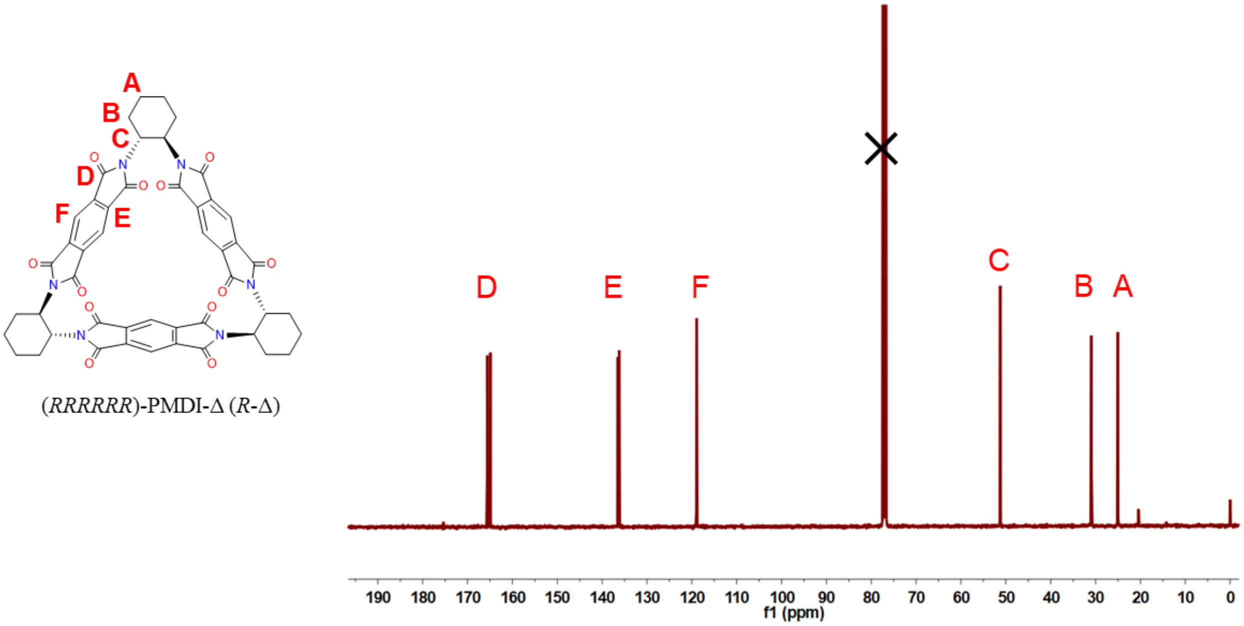

Supplementary Figure 43. ^{13}C-NMR spectrum of *R*-Δ (100 MHz, $CDCl_3$, 298 K).

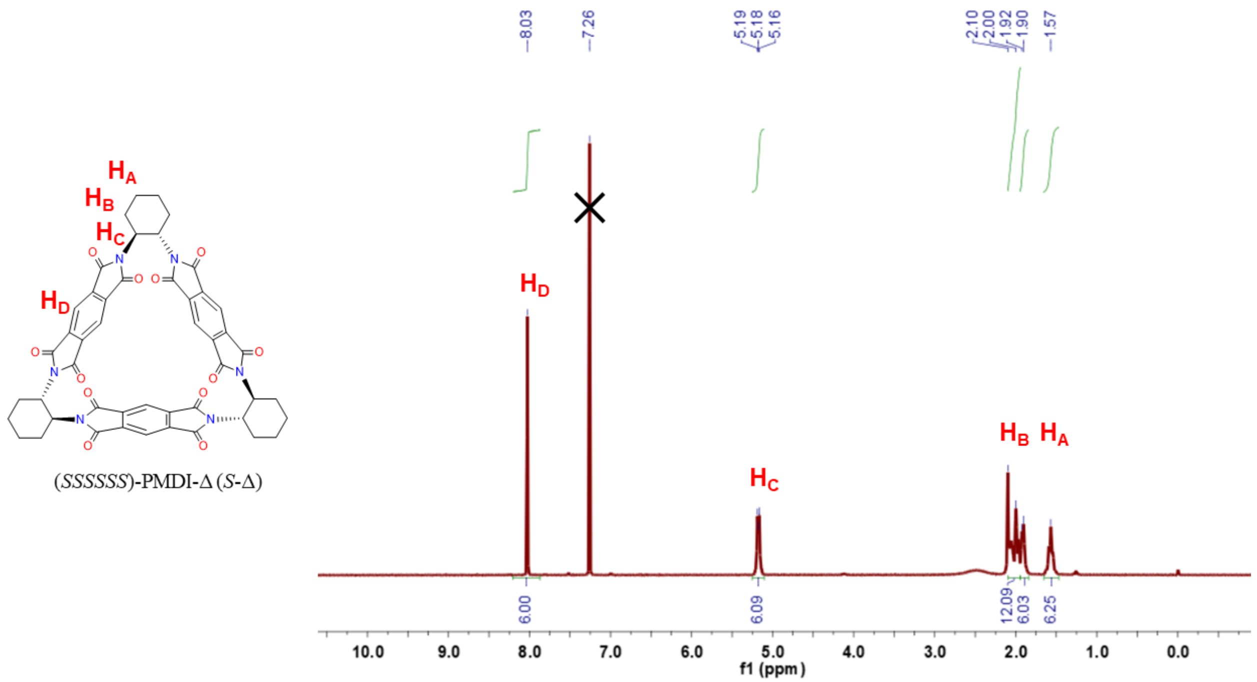

Supplementary Figure 44. ^{1}H-NMR spectrum of *S*-Δ (400 MHz, $CDCl_3$, 298 K).

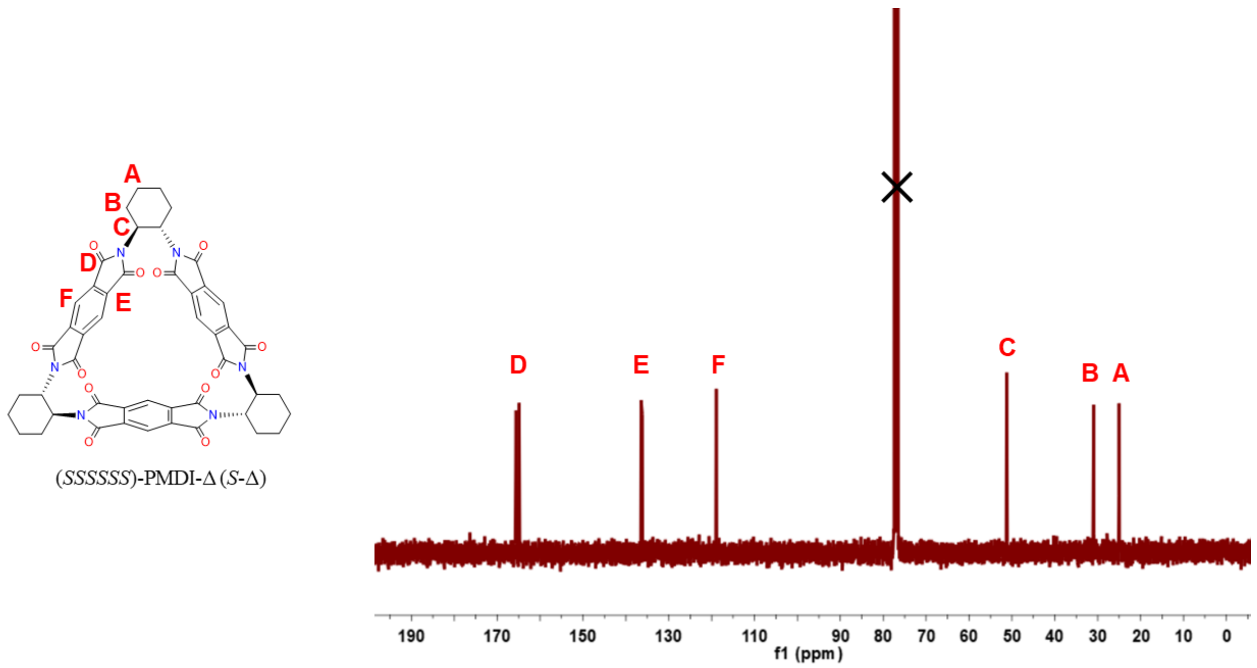

Supplementary Figure 45. ^{13}C-NMR spectrum of *S*-Δ (100 MHz, $CDCl_3$, 298 K).

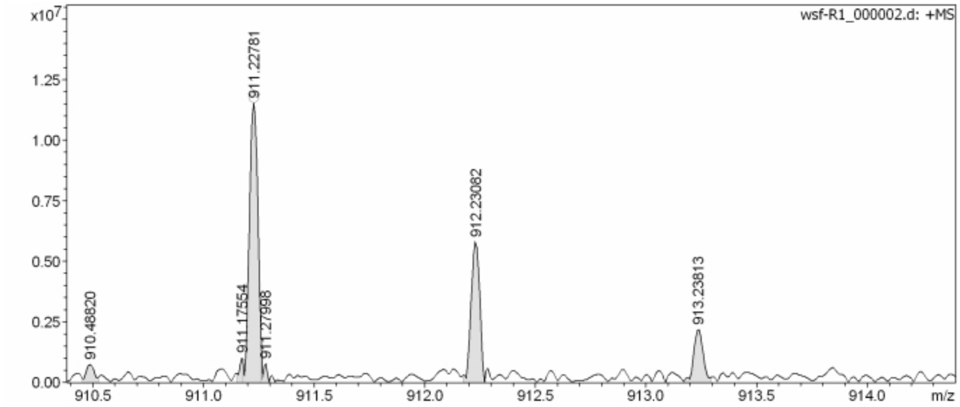

Meas. m/z	#	Ion Formula	Score	m/z	err [ppm]	Mean err [ppm]	mSigma	rdb	e⁻ Conf	N-Rule
911.227810	1	C48H36N6NaO12	100.00	911.228341	-0.6	0.1	28.3	33.5	even	ok

Supplementary Figure 46. The HR-ESI-MS spectrum of *R*-PMDI-Δ.

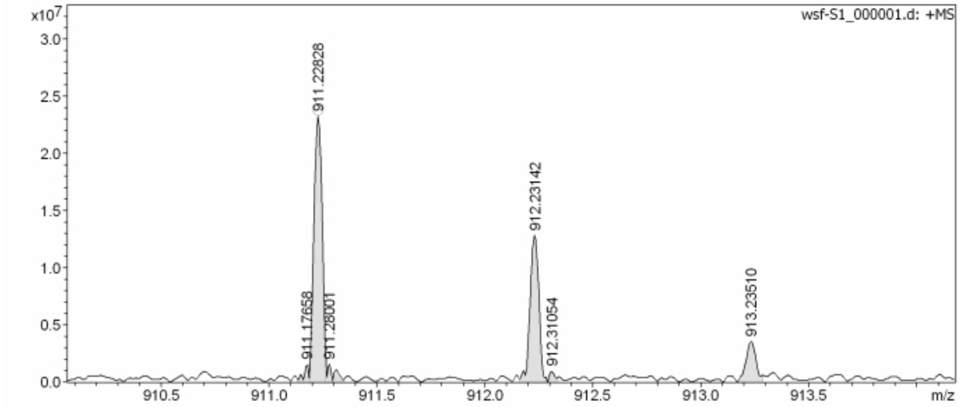

Meas. m/z	#	Ion Formula	Score	m/z	err [ppm]	Mean err [ppm]	mSigma	rdb	e⁻ Conf	N-Rule
911.228282	1	C48H36N6NaO12	100.00	911.228341	-0.1	-0.0	19.8	33.5	even	ok

Supplementary Figure 47. The HR-ESI-MS spectrum of *S*-PMDI-Δ.

11. Supplementary References

1 Kim, D. J. *et al.* Redox-Active Macrocycles for Organic Rechargeable Batteries. *Journal of the American Chemical Society* **139**, 6635-6643, doi:10.1021/jacs.7b01209 (2017).

2 Germán, L., Cuevas, J. M., Cobos, R., Pérez-Alvarez, L. & Vilas-Vilela, J. L. Green alternative cosolvents to N-methyl-2-pyrrolidone in water polyurethane dispersions. *RSC advances* **11**, 19070-19075 (2021).

3 Reichardt, C. Solvatochromic dyes as solvent polarity indicators. *Chemical reviews* **94**, 2319-2358 (1994).

4 Bildyukevich, A. *et al.* Effect of the solvent nature on the structure and performance of poly (amide-imide) ultrafiltration membranes. *Journal of Materials Science* **55**, 9638-9654 (2020).

5 Gupta, S. Viscometry for liquids. *Cham: Springer International Publishing* (2014).

6 Frisch, M. J. *et al*. Gaussian 09, Revision D.01, Gaussian, Inc., Wallingford CT, 2013.

7 Gillgren, H. *et al.* Morphology and molecular conformation in thin films of poly-γ-methyl-L-glutamate at the air-water interface. *Langmuir* **18**, 462-469, doi:10.1021/la011143h (2002).

8 Ohira, A. *et al.* Versatile helical polymer films: Chiroptical inversion switching and memory with re-writable (RW) and write-once read-many (WORM) modes. *Advanced Materials* **16**, 1645-+, doi:10.1002/adma.200400470 (2004).

9 Gottarelli, G., Lena, S., Masiero, S., Pieraccini, S. & Spada, G. P. The use of circular dichroism spectroscopy for studying the chiral molecular self-assembly: An overview. *Chirality* **20**, 471-485, doi:10.1002/chir.20459 (2008).

10 Davidsson, A., Norden, B. & Seth, S. Measurement of oriented circular-dichroism. *Chemical Physics Letters* **70**, 313-316, doi:10.1016/0009-2614(80)85341-3 (1980).
